# Neuroprotective Potential of the Edible Seaweed *Pterocladiella capillacea*: Bioactivity Coupled LC-MS/MS, Metabolomics Annotation, Validation, and Network Pharmacology

**DOI:** 10.3390/nu18193210

**Published:** 2026-09-29

**Authors:** Meicai Yue, Longjian Zhou, Wanrong He, Bin Li, Zhiyou Yang, Yayue Liu, Lingyun Chen, Caiyi Chen, Yi Zhang

**Affiliations:** 1 Guangdong Provincial Key Laboratory of Aquatic Product Processing and Safety, Guangdong Provincial Engineering Laboratory for Marine Biological Products, Guangdong Provincial Engineering Technology Research Center of Seafood, Shenzhen Institute of Guangdong Ocean University, Zhanjiang Municipal Key Laboratory of Marine Drugs and Nutrition for Brain Health, Research Institute for Marine Drugs and Nutrition, College of Food Science and Technology, Guangdong Ocean University, Zhanjiang 524088, China; 19148020452@163.com (M.Y.); zhoulongjian@gdou.edu.cn (L.Z.); superbb1218@163.com (W.H.); libin@fuweiyichao.com (B.L.); zyyang@gdou.edu.cn (Z.Y.); yayue_liu@163.com (Y.L.); 13534915215@163.com (C.C.); 2 Collaborative Innovation Center of Seafood Deep Processing, Dalian Polytechnic University, Dalian 116034, China; 3 BGI Research, Shenzhen 518083, China

**Keywords:** Alzheimer’s disease, neuroprotection, *Pterocladiella capillacea*, activity-guided separation, metabolomics

## Abstract

**Background:** Alzheimer’s disease (AD) is a complex neurodegenerative disorder with a global impact, highlighting an urgent need for effective dietary therapeutic agents for the development of brain-healthy functional foods and medicines. This study focuses on the edible red alga *Pterocladiella capillacea* from Zhanjiang, China, investigating its anti-neuronal injury activity, active principles, and underlying mechanisms. **Methods:** Based on antioxidant and neuroprotection evaluations and column fractioning, bioactivity-coupled liquid chromatography-tandem mass spectrometry (Bio-LC-MS/MS) was employed to recognize the neuroprotective compounds, which were further annotated by comprehensive metabolomics analysis. The annotated compounds were validated by LC-MS/MS and bioassay. Computational prediction techniques were further used to reveal their mechanism of action. **Results:** The study demonstrated that *P. capillacea* extract and fractions exhibited significant antioxidant and anti-neuronal injury activities. Among the annotated and MS-validated potential compounds, erucamide standard exhibited the strongest neuroprotective effect, maintaining cell viability above 85% at low concentrations. Network pharmacology and molecular docking revealed that erucamide may exert neuroprotective effects by modulating the AKT1 and BCL2 targets. ADMET prediction suggested a low risk of central side effects. **Conclusions:** The neuroprotective potential of *P. capillacea* provides a primary foundation for new brain-healthy functional foods and drug development in future.

## 1. Introduction

Alzheimer’s disease (AD) is the most common age-related, severe, and irreversible neurodegenerative disorder, and has become a major challenge, seriously affecting the health and quality of life of the elderly population [1]. The main clinical manifestations are cognitive dysfunction, significant memory loss, feeling emotionally out of control, and loss of motor ability [2].

Although extensive research has been conducted on AD, focusing on genetic factors [3], β-amyloid (Aβ) hypothesis [4], Tau protein abnormal phosphorylation hypothesis [5], oxidative stress hypothesis [6], etc., its pathogenesis remains unclear due to the high complexity of the disease, and no effective and safe treatments and drugs have been found.

At present, the drugs for the treatment of AD are mainly symptomatic relief, which cannot fundamentally reverse the course of the disease and some drawbacks are unavoidable. Traditional cholinergic drugs cause side effects such as gastrointestinal discomfort, dizziness, and cardiovascular abnormalities [7]. Although new targeted drugs like expensive Aβ monoclonal antibodies can slightly delay disease progression in a subset of patients, they may have serious adverse reactions such as cerebral edema and intracranial hemorrhage [8]. Importantly, elderly patients suffer from a heavy burden on the liver and kidneys and poor compliance with long-term use, which further hinder the effectiveness of the drugs.

Functional foods contain rich natural active ingredients, with high safety and few side effects, which are low in cost, have good adherence, and are suitable for long-term consumption. They can comprehensively modulate brain health through multiple pathways such as antioxidation, anti-inflammatory, and neuroprotection [9]. They can be used not only for early prevention and intervention for high-risk groups, but also for improving cognition and quality of life. Moreover, they can assist drug therapies in cognition improvement [10]. Thus, in the context of global population aging and the increasing incidence of AD, it is important to discover dietary bioactive compounds with multi-target properties and low toxicity for the development of new effective functional foods and drugs. In this regard, marine food resource, especially many marine edible algae with rich bioactive compounds and sustainable biomass, will make a great contribution [11].

Seaweeds belonging to the order of Gelidiales are important natural raw materials in the food industry and daily diet. Their main use is to extract agar, which is widely used in jelly, soft candy, ice cream, cans and beverage processing as a gel, thickener, stabilizer and clarifier [12,13]. In terms of daily diet, *Gelidium amansii* is widely used for salads, sea jelly, and soup for, among other things, its characteristics of low fat, high fiber and crisp taste. In addition, Gelidiales seaweed is rich in sulfated polysaccharides, phycobiliproteins, polyphenols and other active ingredients, which have a potential neuroprotective effect on AD [14]. For example, sulfated polysaccharide from *G. pristoides* has been shown to significantly inhibit Zn-induced neurodegeneration in rat hippocampal neuron cells (HT-22) and prevent Zn-triggered late apoptosis and necrosis [15]. As a functional raw material from natural marine sources, Gelidiales seaweeds are safe and suitable for long-term dietary intervention, which can be used as an important method for early prevention and auxiliary intervention of AD.

*Pterocladiella capillacea* is a kind of macroalga belonging to the order Gelidiales and has important economic value [16]. This species mainly grows on the low intertidal rocky coast of tropical and subtropical waters. It flourishes from late spring to late summer [17]. In Guangdong, China, this seaweed has long been consumed as a traditional food and medicinal material. As a kind of nutrient-rich macroalga, a series of studies on its medicinal value have shown that *P. capillacea* is rich in algal polysaccharides, polyunsaturated fatty acids, carotenoids, taurine, and other functional components. It has thus been recognized as an important source of pharmaceutical and nutraceutical materials, showing broad application potential [18]. Wang’s study found that the active components isolated from *P. capillacea* have strong xanthine oxidation inhibitory activity and modest anti-inflammatory activity [19]. De Alencar et al. reported that the 70% ethanol extract of *P. capillacea* had considerable antioxidant capacity, especially in ferrous ion chelation, as well as bacterial agglutination activity on a variety of pathogenic bacteria (including drug-resistant bacteria) [20]. Osman reported that its acetone extract showed good antibacterial activity [21]. In addition, Ismail analyzed the biological characteristics of *P. capillacea* bioactive substances such as sulfated polysaccharides, fatty acids and lectins and found that the active extracts of *P. capillacea* had anti-inflammatory, antioxidant, anticoagulant, antibacterial, antifungal and antifouling activities [22]. However, there is no report on the neuroprotective activity of *P. capillacea* and its related components yet.

In the study of bioactive natural products for functional foods or drugs, the characterization of active molecules is the core foundation for in-depth mechanism study. However, traditional systematic phytochemical isolation/structural elucidation or stepwise bioactivity-guided isolation are extremely time-consuming and inefficient. Bioactivity-coupled liquid chromatography-tandem mass spectrometry (Bio-LC-MS/MS) is a rapid high-throughput analysis strategy based on the correlation between biological activity and mass spectrometry information of small molecules in the extracts. It recognizes the bioactive small molecules by calculating the change in ion abundance in the sample caused by the combination or reaction of small natural product molecules (ligands) in extracts with biological targets (e.g., enzymes or free radicals) [23]. Or it identifies the active substances by calculating the correlation between the biological activity of continuous component samples and the ionic abundances of individual substances in each sample [24]. Bio-LC-MS/MS has the advantages of simple operation, high sensitivity, rapid screening, and low sample amount need, and can be widely used in the screening of active substances in natural products. The quick localization of bioactive compounds in complex natural extracts by Bio-LC-MS/MS, when combined with metabolomics and network pharmacology approaches, will accelerate the discovery of useful biomolecules from natural resources and the development of functional foods and drugs.

This study aimed to efficiently investigate the neuroprotective potential of the edible seaweed *P*. *capillacea* (collected from Zhanjiang coastal waters in the South China Sea) for use as functional foods or therapeutics. For this purpose, antioxidant and neuroprotective bioassays, quick column fractioning, Bio-LC-MS/MS followed by metabolomics annotation, authenticated samples’ chemical/biological validation, and prediction were systematically employed and are reported in this study.

## 2. Materials and Methods

### 2.1. Experimental Materials, Cell Line Sources, and Instruments

*P. capillacea* was purchased from Naozhou Island, Zhanjiang City, China, in March 2025 and morphologically identified by Prof. Enyi Xie in the College of Fisheries, Guangdong Ocean University. Mouse hippocampal neuron cells (HT-22) were purchased with accession number GNM47 from the Cell Resource Center, Shanghai Academy of Biological Sciences, Chinese Academy of Sciences.

In this study, we used a C18 solid phase extraction column (Foshan Kabadi Trading Co., Ltd., Foshan, China); erucamide, hexadecanamide, and arachidonic acid (TargetMol Chemicals Inc., Shanghai, China); oleamide (Beijing Solarbio Science & Technology Co., Ltd., Beijing, China); high-glucose Dulbecco’s Modified Eagle Medium (DMEM) and penicillin/streptomycin solution (Grand Island Biological Company, Shanghai, China); fetal bovine serum (FBS) (Zeta Corporation, Arcadia, CA, USA); 2,2-Diphenyl-1-picrylhydrazyl (DPPH) (Shanghai Yuanye Biotechnology Co., Ltd., Shanghai China); 3-(4,5-dimethylthiazol-2-yl)-2,5-diphenyltetrazolium bromide (MTT) (Macklin Biochemical Technology Co., Ltd., Shanghai, China); and ethyl acetate, methanol, dimethyl sulfoxide, and n-hexane (Guangdong Guanghua Technology Co., Ltd., Shantou, China, analytical grade). We also used a Waters ACQUITY UPLC I-Class System (Waters Corporation, Milford, MA, USA) coupled to a Thermo Fisher Q Exactive HFX mass spectrometer (Thermo Fisher Scientific, Waltham, MA, USA); Bio-Tek Epoch 2 (Agilent Technologies, Inc., Winooski, VT, USA); and Echo RVL-1000-M inverted epifluorescence microscope (Echo, Lake Zurich, IL, USA).

### 2.2. P. capillacea Extraction and Separation

*P. capillacea* was dried naturally and smashed. Ethyl acetate was used as the extraction solvent, and the ratio of material to liquid was 1:10 (g/mL). The crude ethyl acetate extract was obtained by ultrasonic extraction and then condensation in vacuum under room temperature. The ethyl acetate crude extract was desalted with dichloromethane:methanol (1:1, *v*/*v*), dissolved in methanol, and applied on a C18 solid phase extraction column, and then eluted stepwise by acetonitrile:water (10:90, 25:75, 40:60, 55:45, 70:30, 85:15, 100:10, *v*/*v*), acetone, and dichloromethane:methanol (1:1, *v*/*v*) to yield fractions 1–10.

### 2.3. DPPH Free Radical Scavenging Experiment

The ethyl acetate crude extract was dissolved in dimethyl sulfoxide (DMSO) to obtain final concentrations of 0.1, 0.5, 1, 2.5, 10 mg/mL (final concentration). The experiment was carried out according to the following protocol: sample group (*A*1): 50 μL sample solution, 50 μL DPPH solution; sample control group (*A*2): 50 μL sample solution, 50 μL methanol; blank group (*A*3): 50 μL DMSO, 50 μL DPPH solution; blank control group (*A*4): 50 μL DMSO, 50 μL methanol. Three replicates were set. Then, the absorbance at 517 nm was measured by a microplate reader after standing in darkness for 30 min. Vitamin C was used as a positive control. The DPPH free radical scavenging rate was calculated as follows:

(1) $$Clearance\;(\%)=\frac{\left[\left(A_{3}-A_{4}\right)-\left(A_{1}-A_{2}\right)\right]}{\left(A_{3}-A_{4}\right)}\times 100\%$$

### 2.4. Cell Modeling and Toxicity Test

The complete medium of HT-22 cells was composed of an 89% basal medium, 10% fetal bovine serum and 1% penicillin/streptomycin mixture. The cells were cultured in an incubator at 37 °C and 5% CO_2_. The *P. capillacea* samples (crude extract and fractions) were prepared in fresh, serum-free DMEM to final concentrations of 2.5, 5, and 10 μg/mL. Aβ_25–35_ was prepared in the same medium to final concentrations of 5, 10, 20, and 40 μM.

The MTT method was used to evaluate the cytotoxicity of HT-22 cells caused by Aβ_25–35_ and *P. capillacea* samples (the ethyl acetate crude extract and its fractions) [25]. First, the logarithmic phase cells with a confluence of 80–90% were digested and counted. Second, the cell concentration was adjusted to 1 × 10^4^ cells/mL with complete medium, and inoculated into 96-well plates at a volume of 100 μL per well and cultured in the incubator for 24 h. Then, the supernatant was discarded, the samples or Aβ_25–35_ at the specified final concentration were added, and the culture was continued for 24 h. Finally, the cell viability was detected by MTT assay. Then, 90 μL DMEM and 10 μL MTT solution (5 mg/mL) were added. After incubation for 4 h, the supernatant was discarded, 100 μL DMSO was added to each well, and the plates were shaken at 440 r/min for 10 min to fully dissolve the formazan crystals. The absorbance was measured at 490 nm using a microplate reader. Furthermore, after the experiment, the cellular morphology of HT-22 cells in all groups, including the blank group, model group, and experimental groups, was observed under bright-field conditions using an inverted fluorescence microscope to assess the morphological changes induced by Aβ_25–35_ and extract treatment.

### 2.5. Determination of Anti-Neuronal Injury Activity

In this experiment, the MTT assay was used to evaluate Aβ_25–35_-induced cytotoxicity in HT-22 cells and the protective effects of the *P. capillacea* samples against this injury. The cell concentration was adjusted to 1 × 10^4^ cells/mL with complete medium, and inoculated into a 96-well plate at a volume of 100 μL per well and cultured in the incubator for 24 h. The cells were first exposed to 20 μM Aβ_25–35_ for 24 h to induce injury. Subsequently, the treatment groups were treated with *P. capillacea* samples at the specified concentrations, while the blank and model groups received 100 μL of DMEM. All groups were then incubated for an additional 24 h. Finally, the cell viability was detected by MTT assay, and the absorbance value at 490 nm wavelength was detected by a microplate reader.

### 2.6. Bio-LC-MS/MS and Metabolomics Analysis

For Bio-LC-MS/MS analysis (the correlation analysis between ion abundance of specific metabolites and bioactivity), the recovery rate produced by each *P. capillacea* fraction at a specified concentration in Aβ_25–35_-injured cells and the fractions’ MS1-MS2 data were necessary.

The recovery rates were calculated using the original cell viability data as follows:

(2) $$Recovery\;rate\;\left(\%\right)=\;\frac{C_{1}-C_{m}}{C_{0}-C_{m}}\times 100\%$$

where *C*_0_, *C*_1_, and *C_m_* represent the cell viability of the blank group, the treatment group, and the model group, respectively.

For mass data acquisition, the *P. capillacea* fractional samples were analyzed on a Waters ACQUITY UPLC I-Class System coupled to Thermo Fisher Q Exactive HFX mass spectrometer. Chromatographic separation was performed on a Waters ACQUITY UPLC BEH RP18 C18 reversed-phase column (2.1 × 100 mm, 1.7 μm). The injection volume was 5 μL, the sample concentration was 0.2 mg/mL for each sample, the column temperature was 45 °C, and the flow rate was 0.35 mL/min. Gradient elution procedure: 2% B (0–1 min), 2–98% B (1–6 min), 98% B (6–8 min), 98–2% B (8–8.1 min), 2% B (8.1–10 min). In positive ion mode, ultrapure water (containing 0.1% formic acid) was mobile phase A, and methanol (containing 0.1% formic acid) was mobile phase B. In negative ion mode, ultrapure water (containing 10 mM ammonium formate) was used as mobile phase A, and 95% methanol/water (containing 10 mM ammonium formate) was used as mobile phase B.

Mass spectrometry analysis was carried out in positive and negative ion modes, using an electrospray ion source (ESI). The scan range was set to mass-to-charge ratio (*m*/*z*) from 70.00 to 1050.00, and all raw data were saved in the .raw file format.

The correlation analysis between MS information and bioactivity comprised the following steps. First, LC-MS/MS mass spectrometry data processing was performed using MS-DIAL to detect and quantify the features (ions with specific retention time, parent mass, and MS/MS spectra) in fractional samples and export feature tables and .mgf files necessary for the next step computation. Second, the Pearson correlation coefficients (cor values) were calculated between the abundances of the specific ions and the corresponding biological activity levels of the samples. The relevant scripts were written in R language and deployed in a Jupyter notebook environment (http://jupyter.org/) [24].

For the metabolomics annotation of the bioactivity highly correlated compounds, a comprehensive annotation strategy was applied by integrating MS-DIAL, MS-FINDER, GNPS (Global Natural Products Social Molecular Networking), manual MS1 and isotopic pattern inspection by Xcalibur 2.2.42 and ChemDraw 2020, and MetFrag Web beta multi-database searching and fragmentation simulation to improve the rationality of structural annotations.

MS-DIAL and MS-FINDER were used to match MS1-MS2 spectra with the experimental and calculated MS spectra of compound databases [26]. The GNPS analysis was performed following the routine workflow [27], and then visualized in Cytoscape_v3.7.2 to construct a molecular network, which provided putative annotations for compounds of interest. Then the Xcalibur software was used to compare the original high-resolution mass and isotopic pattern of the parent ions with the theoretical values calculated by software [28,29]. The MS1-MS2 spectrum information was submitted to the Met-Frag platform with multi-database retrieval to verify and simulate MS2 fragmentation [30]. All the annotated compounds were also searched in commonly used natural product databases (PubChem, COCONUT, NPBS Atlas, etc.) to check their biological source. The final annotations were made based on the overall consideration of these approaches.

### 2.7. LC-MS/MS Validation of Annotated Compounds Using Commercial Standards

The authenticated standards of the annotated compounds, the *P. capillacea* ethyl acetate extract, and related fractions were analyzed using the same LC-MS/MS conditions described above.

### 2.8. Network Pharmacology Prediction

To predict the potential neuroprotective targets and mechanism of erucamide by network pharmacology [31], the SMILES code of erucamide was first uploaded to the Swiss Target Prediction website (http://www.swisstargetprediction.ch) on 27 March 2026 for target prediction. In parallel, Alzheimer’s disease (AD)-related targets were retrieved from the MalaCards database (https://www.malacards.org/) and GeneCards database (https://www.genecards.org/). The targets of erucamide and the AD-related targets were intersected to obtain the overlapping targets, and the Venn diagram was generated using R (v4.5.1) with the ggVennDiagram package to visualize the intersection. The overlapping targets were then imported into the STRING database (https://string-db.org/) to construct the protein-protein interaction (PPI) network. Three topological analysis algorithms (MCC, Degree, and EPC) implemented in Cytoscape (v3.10.4) with the CytoHubba plugin were applied to identify the core hub targets. Further, Metascape (https://metascape.org/) on 28 March 2026, was used for GO and KEGG enrichment analysis.

### 2.9. Molecular Docking

Based on the core targets identified by network pharmacology, the three-dimensional crystal structures of AKT1 (PDB ID: 4GV1, resolution 2.00 Å) and BCL2 (PDB ID: 6GL8, resolution 2.50 Å) were retrieved from the RCSB Protein Data Bank, and the molecular structure of erucamide was constructed and energy-minimized. Protein preparation was performed using AutoDock Tools (v1.5.7), including removal of water molecules and co-crystallized ligands, addition of polar hydrogen atoms, and assignment of Gasteiger partial charges. For the ligand, rotatable bonds were defined and charges were computed, and all structures were saved in PDBQT format. The docking grid boxes were centered on the coordinates of the original co-crystallized ligands to define the active sites. For AKT1, the grid box was set at center_x = −21.09, center_y = −3.72, center_z = 16.75 with dimensions of 25.00 × 31.40 × 27.80 Å. For BCL2, the grid box was set at center_x = 17.19, center_y = 2.65, center_z = 15.65 with dimensions of 25.00 × 25.00 × 25.00 Å. Semi-flexible docking was performed using AutoDock Vina (v1.2.0) [32]. The conformation with the lowest binding affinity was selected for each target. The resulting ligand-protein complexes were visualized and analyzed using PyMOL (v3.1.0) and Discovery Studio Visualizer (v21.1.0) to characterize the binding modes.

### 2.10. Admet

The absorption, distribution, metabolism, excretion and toxicity characteristics of compounds were predicted by using the ADMETlab 3.0 online prediction website (https://admetlab3.scbdd.com/server/evaluationCal) on 28 March 2026 through the SMILES code of the input compounds [33]. The solubility, permeability, bioavailability, distribution volume, blood-brain barrier permeability, hepatotoxicity, and skin sensitization of the compounds were analyzed.

### 2.11. Statistical Analysis

All data were expressed as mean ± standard error of the mean (SEM). GraphPad Prism (version 8.4.3) was used to analyze the experimental data. One-way ANOVA was used to analyze the data of more than two groups. *p* < 0.05 was considered significant.

## 3. Results

### 3.1. DPPH Free Radical Scavenging and Neuroprotective Activities of P. capillacea Extract

In the pathological process of AD, oxidative stress is one of the core driving mechanisms [34], and DPPH free radical scavenging activity is a classic in vitro antioxidant evaluation assay [35]. Natural products with strong DPPH radical scavenging ability may effectively neutralize excessive reactive oxygen species (ROS) in vivo, interrupt oxidative stress cascades, and protect neurons.

In the DPPH scavenging assay (Figure 1), *P. capillacea* ethyl acetate extract exhibited dose-dependent antioxidant activity with a clearance rate over 70% at the concentration of 10 mg/mL and an IC_50_ of 1.05 ± 0.15 mg/mL.

On this basis, the extract was further evaluated for its neuroprotective effect. To establish the neuronal injury model, 5 to 40 μM Aβ_25–35_ was tested for its damage to HT-22 cells. The cell viability decreased along the Aβ_25–35_ concentrations. Among them, 20 μM Aβ_25–35_ caused moderate damage with cell viability decrease (65%) suitable for modeling and the cells showed typical shortened synapses compared with the blank (Figure 2A,B), which was consistent with a related report [36]. Thus, 20 μM Aβ_25–35_ was determined for modeling.

The *P*. *capillacea* extract was assessed for its toxicity to HT-22 cells and protection to 20 μM Aβ_25–35_-injured HT-22 cells. Within the concentration range of 2.5 to 10 μg/mL, no toxicity was observed for the extract (Figure 2C) while the neuronal damage induced by Aβ_25–35_ was dose-dependently reversed by the extract (Figure 2D). Even at the lowest dose, the extract can totally recover the damage. The cells also recovered their synapses under the treatment (Figure 2A).

### 3.2. Fractioning of P. capillacea Extract on Column, LC-MS/MS Analysis and Neuroprotection Evaluation of Post-Column Fractions, and Bioactivity-Coupled LC-MS/MS Calculation

To trace the neuroprotective compounds in *P. capillacea*, the extract was separated on a C18 solid-phase extraction (SPE) column to afford 10 fractions, F1–F10. The base peak chromatograms (BPC) of LC-MS/MS in positive and negative modes (Figure 3A,B) showed that the fractions contained continuous but gradually different constituents with decreasing polarities, providing a foundation for next-step bioactivity tracing and correlation calculation between bioactivity and metabolite abundances.

In the toxicity evaluation, all ten post-column fractions showed no toxicity to HT-22 cells at the concentrations of 2.5–10 μg/mL (Figure 4A). On this basis, the fractions were screened for neuroprotective activities, and their recovery rates against injury in HT-22 cells were also calculated. Generally, all the fractions exhibited activity to different extents across the tested concentrations (Figure 4B and Figure 5). Among them, F2, F3, F6, and F8 showed dose-dependent protection within the concentration range of 2.5–10 μg/mL; F1, F5, and F9 showed the highest activity at 5 μg/mL; and F4, F7, and F10 exhibited the strongest activity at 2.5 μg/mL.

Based on the bioactivity values (i.e., recovery rates) of different fractions at specific dose and metabolites’ ion abundance across the fractions (for all samples, LC-MS/MS data were collected at the same concentration as described above), the Pearson correlation coefficient (i.e., the cor value in Table 1) between the ion abundance of each metabolite and the bioactivity value was calculated.

### 3.3. Metabolome Annotation of Potential Neuroprotective Molecules

According to the results of the Bio-LC-MS/MS, MS-DIAL, MS-FINDER, and GNPS were used to annotate the potential neuroprotective molecules, while Xcalibur, ChemDraw, and Met-Frag were used to verify the rationality of the mass spectrometry data (Figure 6 and Figures S1–S3). All annotation results were searched against natural product databases to trace their biological source reports (Table 1). Ultimately, four compounds with high cor values and remarkable abundances were structurally annotated, including hexadecanamide, oleamide, erucamide, and arachidonic acid. Accordingly, all four annotated compounds were subjected to standard-based validation.

### 3.4. Verification of Annotated Compounds by LC-MS/MS with Commercial Standards

To verify the reliability of the Bio-LC-MS/MS annotation results, four authentic standards, namely, hexadecanamide, oleamide, erucamide, and arachidonic acid, were procured and analyzed under identical LC-MS/MS conditions alongside the corresponding SPE fractions and extract E1 (ethyl acetate extract of *P. capillacea*). Based on the comparison of the base peak chromatogram (BPC) of E1, the extracted ion chromatograms (EICs) and MS1/MS2 spectra of the four target compounds (Figure 7), combined with systematic spectral comparisons against the authentic standards, as well as the consistent retention times, precursor ion masses, and MS2 fragmentation patterns observed between the SPE fractions and the standards (Figure 8 and Figures S4–S6), the results fully demonstrate that these four compounds are naturally occurring constituents of *P. capillacea*.

### 3.5. Evaluation of Neuroprotection Activity of Annotated Compounds

On the basis of LC-MS/MS validation, the four compounds were further evaluated for their toxicity and neuroprotective activity. The results showed that the four compounds had almost no toxic effect on HT-22 cells at a dose of 0.25─5 μM (Figure 9A–D). For the HT-22 cells injured by 20 μM Aβ_25–35_, erucamide effectively alleviated neuronal damage in a generally dose-dependent manner, oleamide and hexadecanamide also exhibited weak neuroprotective activity, while arachidonic acid did not display significant neuroprotection (Figure 9F–H). Thus, among the annotated potential compounds, erucamide contributes most to the neuroprotective effect of *P. capillacea*, considering it has the strongest activity and high abundance.

### 3.6. Key Target Prediction and Molecular Docking for the Neuroprotective Mechanism of Erucamide

In total, 101 potential targets of erucamide were predicted through the Swiss Target Prediction website. These targets were classified into six functional categories: receptor, enzyme, transcriptional regulation, transport binding, signal regulation, and apoptosis metabolism (Figure 10A). By constructing a Venn diagram, the potential targets of erucamide and AD-related targets (1571 collected from AD-related databases) were mapped and intersected, and 31 overlapping targets were obtained (Figure 10A,C).

In order to investigate the potential effects of the intersection targets on AD treatment, they were imported into the STRING database for analysis and visualized in Cytoscape. A total of 31 nodes are shown in the PPI network diagram (Figure 10B). Furthermore, three topological analysis algorithms (MCC, Degree, and EPC) were used to rank and visualize the network nodes. Six core proteins were selected according to their scores from high to low: EGFR, HIF1A, HSP90AA1, BCL2, AKT1, and PPARG (Figure 10D–F). Based on the three algorithms, BCL2 and AKT1 were predicted to be the top-ranked hub nodes in the network. Further enrichment analysis was therefore performed with a focus on BCL2 and AKT1 to explore their potential roles. However, these findings are derived from computational network analysis and only suggest potential key targets; they do not establish the necessity of BCL2 or AKT1 as protective mediators, which requires further experimental validation.

The GO results showed that these gene sets play an important role in neural development, cell movement, and signal transduction. They are significantly involved in cell migration, movement regulation and the PI3K/AKT signaling pathway in biological processes, and are key regulators of cell movement and signal transduction (Figure 10G). Although the KEGG bubble diagram showed that the intersection targets of erucamide against neuronal damage were significantly enriched in multiple cancer-related pathways (e.g., pathways in cancer, and prostate cancer), this did not indicate that the drug had a carcinogenic risk (Figure 10H). On the contrary, this reflects the high overlap between AD and tumors in the underlying molecular mechanisms such as cell cycle regulation, apoptosis, oxidative stress, and inflammatory response [41,42]. For example, the enriched EGFR/ErbB signaling pathway and HIF-1 signaling pathway are key networks that regulate cell survival and angiogenesis [43]; the lipid and atherosclerosis pathway is consistent with the potential of erucamide as a fatty acid derivative to regulate neuroimmunity and lipid metabolism [44]. The results suggest that erucamide may play a neuroprotective role in inhibiting neuronal apoptosis, attenuating neuroinflammation and neurotrophic support by interfering with these hub networks that regulate cell fate and metabolism.

The results of molecular docking showed that the binding affinity of erucamide with AKT1 and BCL2 was −6.01 kcal/mol and −5.29 kcal/mol, respectively, indicating that there was a moderate molecular binding between erucamide and these target proteins. Structural analysis showed that erucamide was precisely embedded in the hydrophobic binding pocket of AKT1 and BCL2 proteins, and its binding mode was synergistically mediated by a variety of non-covalent forces, including van der Waals forces, conventional hydrogen bonds, alkyl interactions, and alkyl-π interactions. These forces together stabilize the conformation of the ligand–protein complex and provide a structural basis for the predicted binding mode of erucamide to AKT1 and BCL2 proteins (Figure 11). However, because erucamide is hydrophobic, such interactions may be largely nonspecific, and direct experimental validation of target engagement (e.g., SPR, MST, ITC, CETSA, or DARTS) is still required to confirm true biological binding.

### 3.7. Erucamide Has Acceptable ADMET and Drug-like Properties

According to the ADMET analysis results, erucamide conforms to the five rules of Lipinski drugs (molecular weight 337.33, hydrogen bond donor number 2), indicating favorable predicted drug-like properties. The Caco-2 cell permeability, PAMPA passive membrane permeability and human intestinal absorption efficiency were rated as excellent, indicating potential bioavailability of oral administration. In terms of central delivery, the risk of P-glycoprotein substrates and inhibitors is extremely low, which can effectively avoid being cleared by efflux transporters and ensure drug exposure in the brain; at the same time, moderate distribution volume helps to maintain stable in vivo exposure, and extremely low autofluorescence interference facilitates subsequent mechanism research. More importantly, its drug-induced neurotoxicity and liver injury risks are at a very low level, which can effectively reduce the hidden dangers of common toxic and side effects in central treatment, and provide useful preliminary support for the future development of erucamide as a nutritional supplement or lead compound for Alzheimer’s disease intervention (Figure 12 and Table 2). However, it should be noted that the predicted low blood-brain barrier permeability of erucamide may limit its direct transport into the brain, which could potentially be addressed by suitable delivery systems. Importantly, all these ADMET predictions remain computational estimates and require experimental pharmacokinetic and toxicological validation in vivo.

## 4. Discussion

In this study, the neuroprotective potential of edible seaweed *P. capillacea* was systematically explored using an integrated technological pipeline of “Bio-LC-MS/MS-metabolomics annotation-validation-network pharmacology analysis”. As the main validated active ingredient, erucamide was elucidated for its potential molecular mechanism of neuroprotection, which provided a basis for the medicinal or nutritional development and neuroprotection-related research of *P. capillacea*.

At present, natural products have become an important source for the development of neuroprotective functional foods or drugs due to their favorable safety and low number of side effects [45,46,47]. *P. capillacea* as a traditional edible seaweed in the order of Gelidiales has considerable natural biomass and its artificial breeding and large-scale cultivation can be promoted by branch regeneration, as reported by Choi [16]. However, neuroprotective potential research on *P. capillacea* is totally absent. The present study pioneers a novel approach for the high-value-added exploitation of this seaweed resource.

Based on Bio-LC-MS/MS technology, a comprehensive structural annotation strategy consisting of different metabolomics annotation tools (MS-DIAL, MS-FINDER, GNPS), manual inspection of MS1 isotopic pattern and MS2 fragmentation simulation by ChemDraw and Met-Frag, and bioresource retrieval in natural product databases, was applied to provide convincing structural annotations. This cross-validation ensured the rationality and accuracy of annotation, which was manifested by the high confirmation rate of LC-MS/MS validation using commercial standards. The solid validation of ingredients laid a foundation for the subsequent bioactivity verification and mechanism research of core active ingredients.

Among the annotated compounds, erucamide showed the strongest neuroprotective activity. This result is also supported by related studies. Kim et al. reported that erucamide can significantly reduce trimethyl tin (TMT)-induced learning and memory deficits in mice, suggesting its preventive effect on AD-related memory impairment. The mechanism may be related to the regulation of cholinergic function [48]. It also displayed antidepressant and anxiolytic-like behavioral effects by regulating the neuroendocrine system (HPA axis), which is manifested in the reduction in stress hormones ACTH and corticosterone [49]. However, its anti-neuronal injury activity and associated mechanism have not been previously reported. This study first clarified the neuroprotective potential of erucamide and enriched its neuropharmacological activity research.

To further elucidate the neuroprotective mechanism of erucamide, network pharmacology prediction and molecular docking simulation were integrated. The docking results predicted that erucamide may form stable interactions with two key proteins, AKT1 and BCL2, suggesting a potential mechanism involving these core targets. However, because erucamide is hydrophobic, such interactions may be largely nonspecific, and direct experimental validation of target engagement (e.g., SPR, MST, ITC, CETSA, or DARTS) is still required to confirm true biological binding. GO functional enrichment analysis further indicated that the target gene set was significantly enriched in the PI3K/AKT signaling pathway and apoptosis-related pathways. These computational predictions raise the possibility that erucamide, possibly through modulating AKT1 and BCL2, may influence the PI3K/AKT pathway, thereby potentially inhibiting neuronal apoptosis and exerting neuroprotective effects. However, it should be noted that direct experimental evidence for effects on Tau pathology was not obtained in this study; any implication of Tau modulation remains a hypothetical downstream possibility requiring future validation. The predicted involvement of the PI3K/AKT pathway is consistent with previous reports that its activation can inhibit neuronal apoptosis and ameliorate neuropathological damage [50]. Indeed, this pathway plays a key role in regulating neuroinflammation, maintaining blood–brain barrier integrity, and promoting neuronal survival; its activation can suppress NF-κB-mediated pro-inflammatory cytokine expression, reduce neuroinflammation, and thereby limit brain injury [51,52]. These observations echo findings with natural products such as berberine, which exert neuroprotective effects via PI3K/AKT activation [53]. To validate whether AKT1 and BCL2 are necessary mediators of erucamide’s neuroprotective effects, future studies should employ pharmacological inhibitors or antagonists (e.g., PI3K/AKT inhibitors such as LY294002 or MK-2206, and BCL2 inhibitors such as ABT-737) and genetic manipulation (e.g., siRNA/shRNA knockdown or CRISPR/Cas9 knockout of AKT1 and BCL2) in the same Aβ_25–35_-induced HT-22 cell model, followed by functional neuroprotection assays. Overall, our computational and enrichment analyses suggest that erucamide may target AKT1, BCL2, and the PI3K/AKT axis, highlighting its potential value in neuroprotection, though these mechanistic hypotheses require experimental verification.

To validate whether AKT1 and BCL2 are necessary mediators of erucamide’s neuroprotective effects, future studies should employ pharmacological inhibitors or antagonists (e.g., PI3K/AKT inhibitors such as LY294002 or MK-2206, and BCL2 inhibitors such as ABT-737) and genetic manipulation (e.g., siRNA/shRNA knockdown or CRISPR/Cas9 knockout of AKT1 and BCL2) in the same Aβ_25–35_-induced HT-22 cell model, followed by functional neuroprotection assays. Such experiments would help establish target engagement and causality, and clarify whether these predicted core targets are truly required for the observed effects.

In drug research and development, ADMET characteristics and drug-likeness are the core indicators for evaluating the drug potential of compounds. This study confirmed that erucamide has acceptable ADMET characteristics and drug-likeness, indicating that the compound has the potential to be further developed as a neuroprotective drug or a functional nutraceutical. Notably, erucamide was detected as an intrinsic constituent of the less-polar fractions isolated from Adriatic marine brown algae *Ericaria crinita* and *Ericaria amentacea* [39].

Through the integration of multiple technologies, this study not only demonstrated the neuroprotective potential of *P. capillacea*, but also revealed the neuroprotective mechanism and pharmaceutical potential of erucamide for the first time. However, there are still some limitations to be improved in future study.

First, subsequent cell experiments, including pharmacological inhibition/antagonism and genetic manipulation (e.g., siRNA/shRNA knockdown or CRISPR/Cas9 knockout), are needed to further verify the regulatory effect of erucamide on AKT1, BCL2, and the PI3K/AKT pathway.

Second, the neuroprotective effect of erucamide is only verified by in vitro experiments on an Aβ-induced cellular model or virtual studies, and it cannot fully recapitulate the complex pathology of Alzheimer’s disease, including Tau pathology, neuroinflammation, synaptic dysfunction, and the in vivo microenvironment, so its in vivo effects remain to be further verified on animal models and by clinical trials. And the actual erucamide content in seaweed or related food products, bioavailability, achievability, and strict safety evaluations are also crucial factors/aspects that should be taken into account in this in vivo exposure or clinical trials for the purpose of functional development.

Specifically, in ADMET prediction, erucamide shows weak BBB penetration ability, which may influence its direct in vivo effect. The actual oral bioavailability and brain exposure of erucamide remain to be determined experimentally. It is possible that a further delivery system like liposome or structural modification is necessary to improve this constraint.

Moreover, though Bio-LC-MS/MS with a comprehensive metabolomics strategy can provide rational structural annotation to possible bioactive compounds, there are still some compounds for which reliable annotations cannot be generated due to the limited spectral data volume in databases. Nevertheless, these unknown compounds also leave space for future discovery of novel bioactive molecules. We also observed that some annotated compounds had higher cor values than erucamide in the coupling calculation but exhibited weaker activity in bioassays. We speculated that these lower active compounds may coexist with other active compounds in fractions, and their concentration distribution pattern coincidently overlap with the bioactivity distribution pattern of the fractions. So, although Bio-LC-MS/MS method can provide useful guidance for bioactive compound speculation/localization, the validation of standards or isolated compounds is still necessary, which may serve as a reference for the application of Bio-LC-MS/MS in natural product research.

In summary, this study clarified the neuroprotective potential of the edible seaweed *P. capillacea* and its active principles, especially erucamide and the related mechanism of action against neuronal damage, providing an important reference for the high-value-added exploration of *P. capillacea*. Future study will focus on the experimental neuroprotective mechanisms, improved brain delivery, and in vivo effect of erucamide as well as in-depth bioactive compound discovery from *P*. *capillacea* and functional food formula or drug development.

## 5. Conclusions

The edible seaweed *P. capillacea* possesses neuroprotective potential derived from active principles including erucamide, providing a primary foundation for new brain-healthy functional foods and drug development in future.

## Figures and Tables

**Figure 1:**
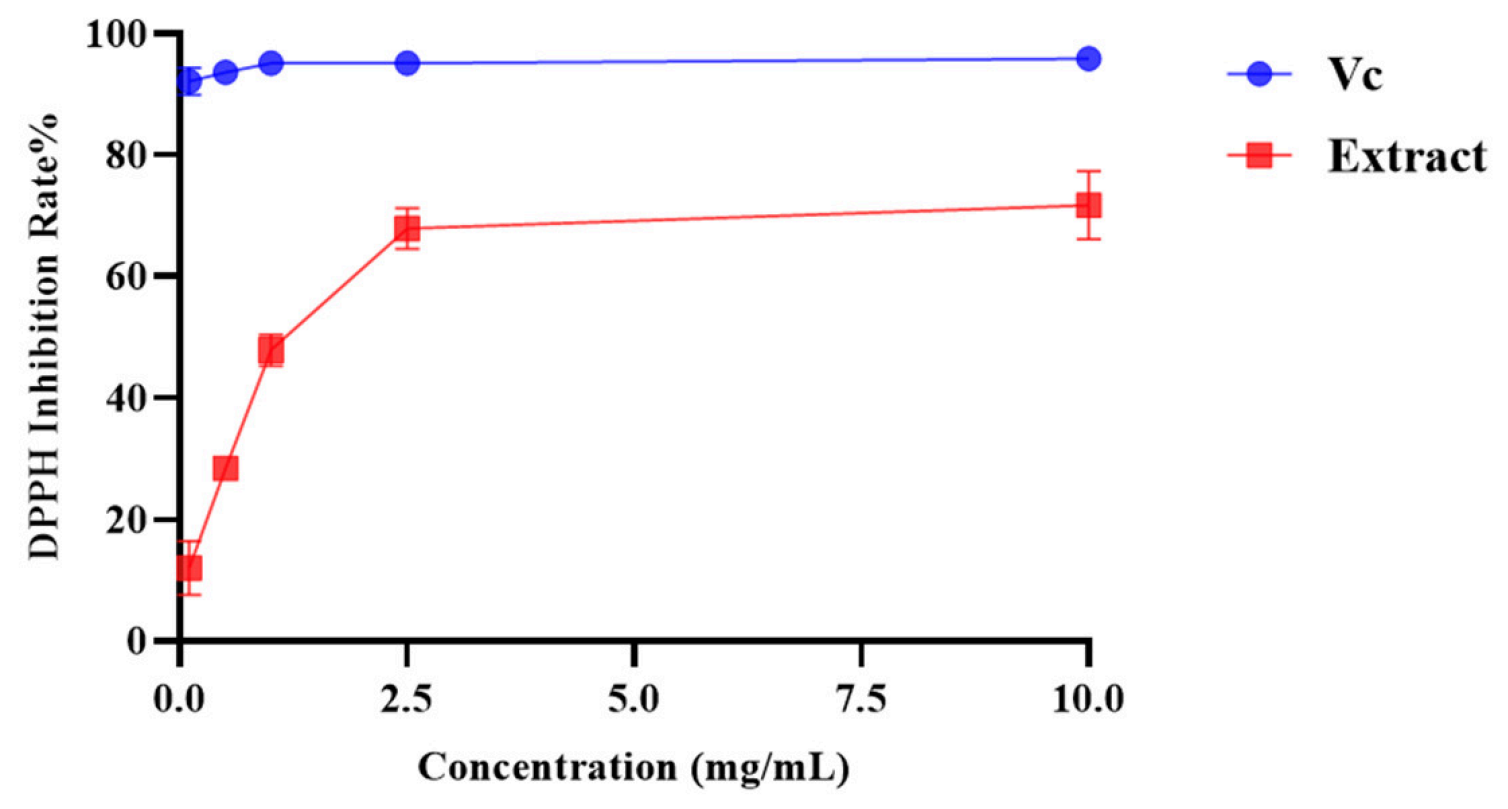
DPPH radical scavenging activity of the *P. capillacea* ethyl acetate extract and positive control V_C_.

**Figure 2:**
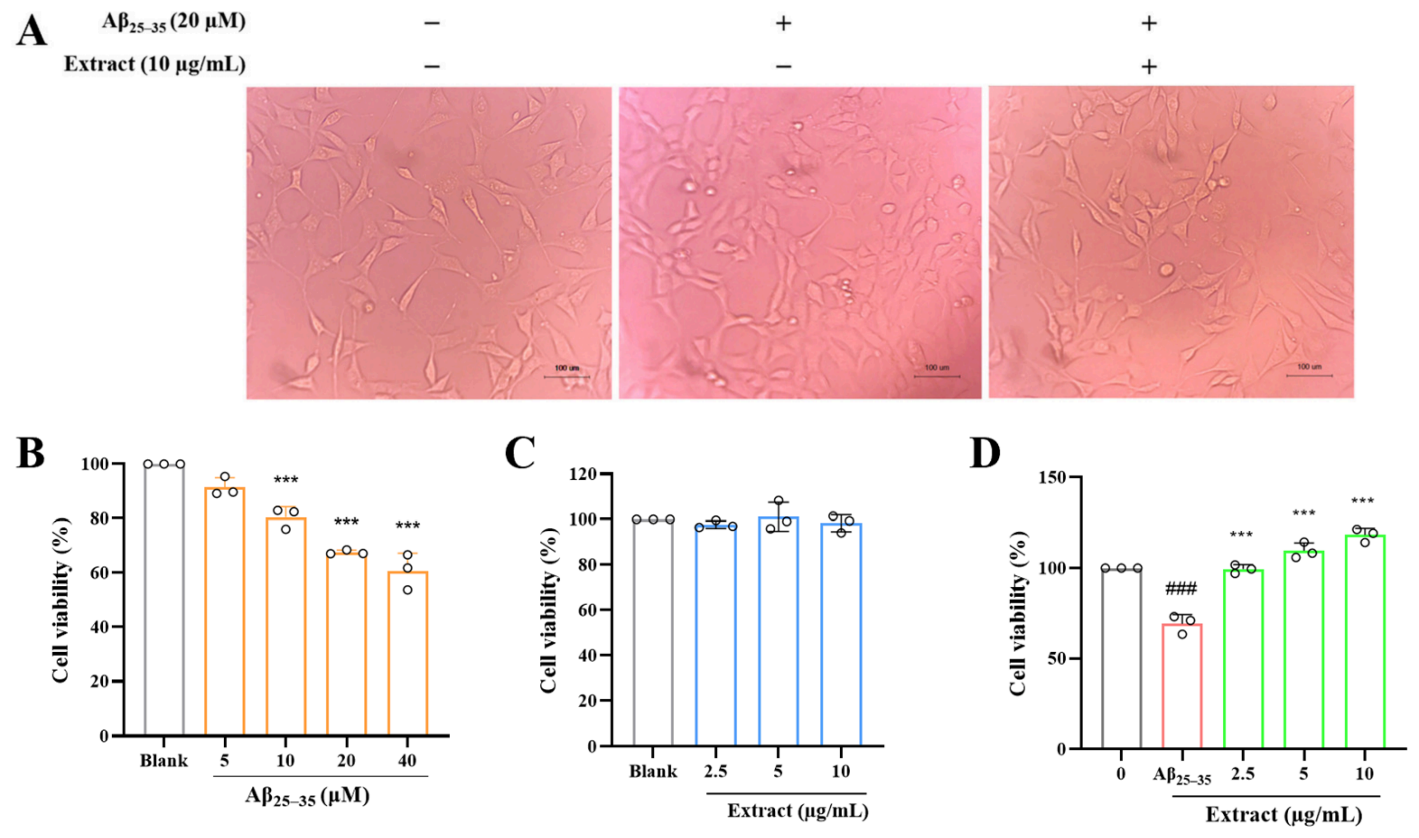
Neuronal injury modeling and the toxicity and neuroprotective activity evaluation of *P. capillacea* ethyl acetate extract. (**A**) The morphology of HT-22 cells treated with Aβ_25–35_ and extract observed under the bright field of an inverted fluorescence microscope. (**B**) Cell viability of HT-22 cells treated with Aβ_25–35_. (**C**) Cell viability of HT-22 cells treated with extract. (**D**) The protective effect of the extract on Aβ_25–35_-induced HT-22 cytotoxicity. “#” indicates a significant difference between the model group and the blank control group (^###^ *p* < 0.001); “*” indicates a significant difference between the model group and the experimental group (*** *p* < 0.001).

**Figure 3:**
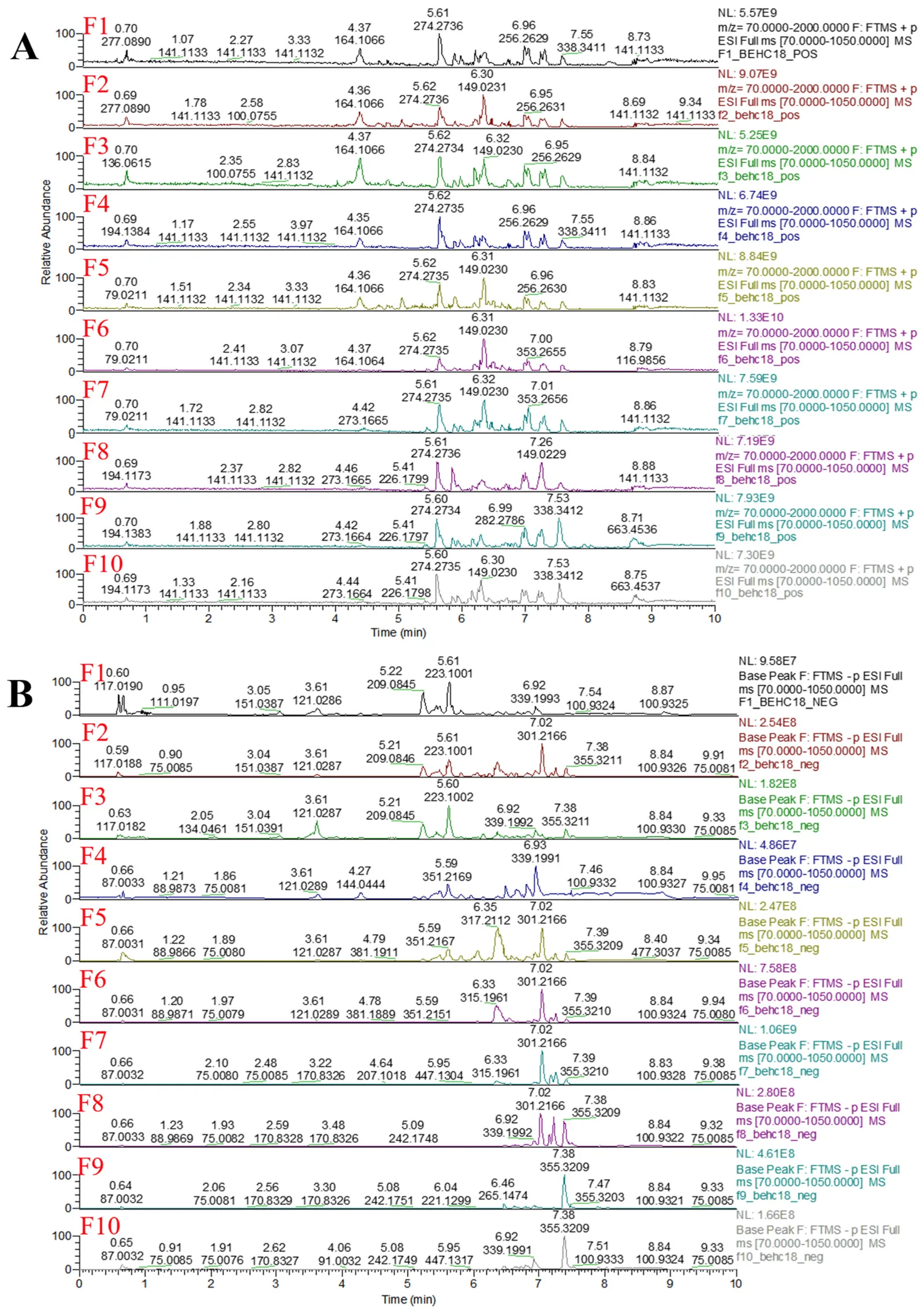
Base peak chromatograms in positive (**A**) and negative (**B**) modes of fractions 1–10 prepared by solid-phase C18 column fractioning of *P. capillacea* ethyl acetate extract. F1–F10 represent components 1–10, respectively.

**Figure 4:**
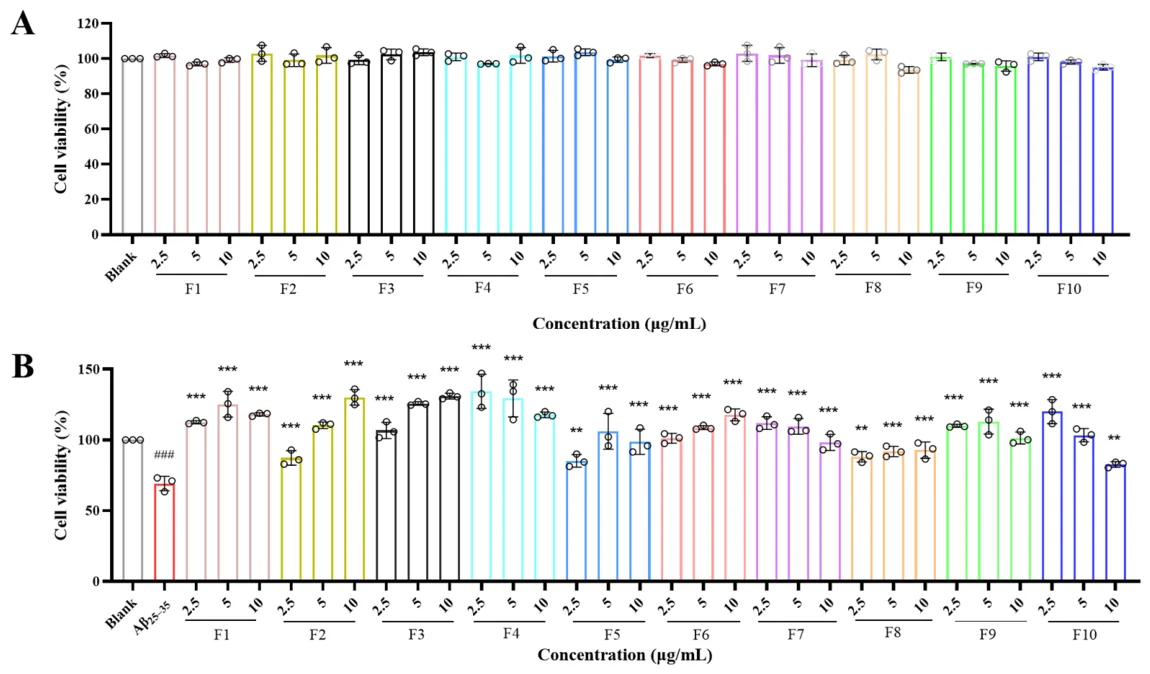
Effects of different *P. capillacea* fractions on Aβ_25–35_ induced neurotoxicity in HT-22 cells: (**A**) cell viability of HT-22 cells treated with *P. capillacea* fractions at different concentrations for 24 h, measured by MTT assay; (**B**) cell viability of HT-22 cells treated with 20 µM Aβ_25–35_ for 24 h, followed by treatment with various *P. capillacea* fractions for an additional 24 h, measured by MTT assay. “#” indicates a significant difference between the model group and the blank control group (^###^ *p* < 0.001); “*” indicates a significant difference compared with the model group (** *p* < 0.01, *** *p* < 0.001).

**Figure 5:**
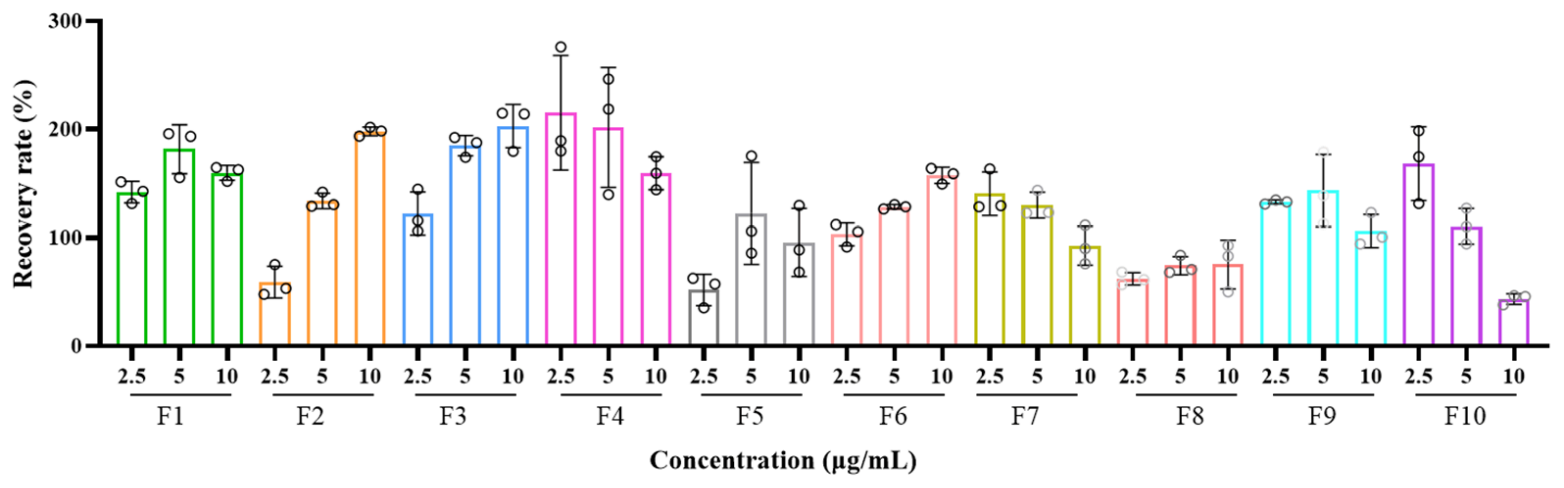
Relative recovery rates of HT-22 cells stimulated by various *P. capillacea* fractions F1–F10.

**Figure 6:**
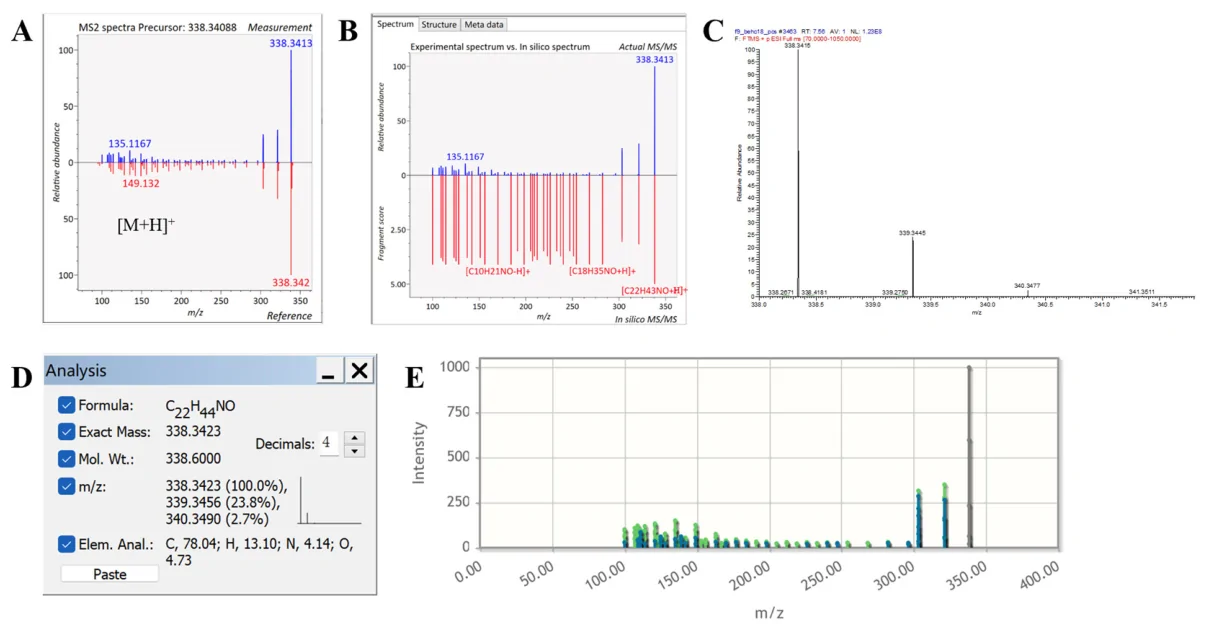
Detailed annotation of erucamide: (**A**) MS2 spectral matching of the compound annotated by MS-DIAL; (**B**) MS2 spectral matching of the compound annotated by MS-FINDER; (**C**) Xcalibur-based analysis of MS1 data and precursor ion isotope pattern fidelity; (**D**) ChemDraw-simulated ion molecular formula calculation; (**E**) MS2 spectral matching of the compound annotated by Met-Frag (in which the blue peaks represent the ones detected in measurement, the green ones represent the theoretical fragments based on structure, while the gray one represents the residue of precursor ion).

**Figure 7:**
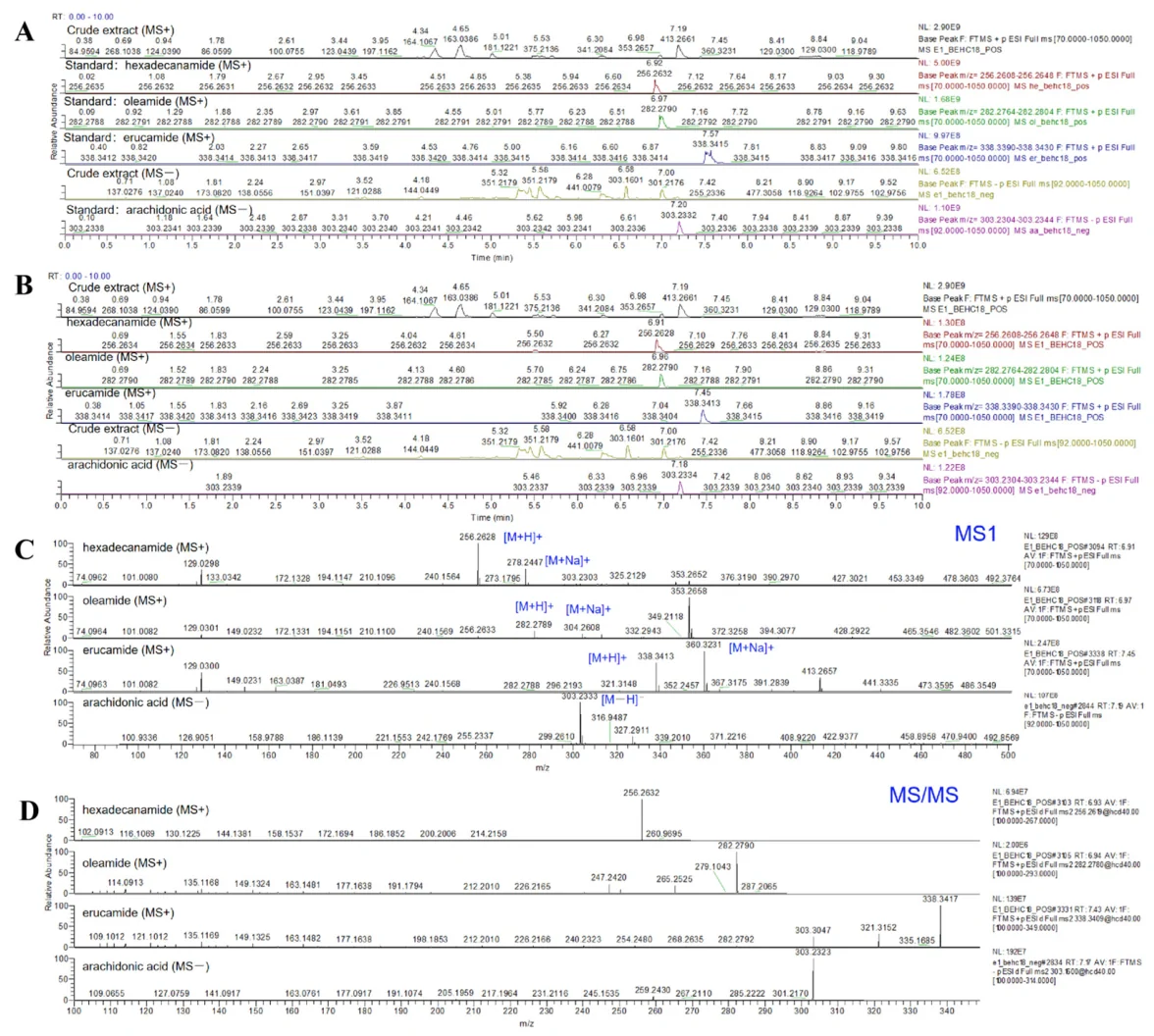
LC-MS/MS spectra for qualitative analysis of the extract and reference standards: (**A**) overlay of base peak chromatograms (BPC) of the extract and the extraction ion chromatograms (EIC) of the four authentic standards, namely, hexadecanamide, oleamide, erucamide, and arachidonic acid; (**B**) overlay of BPC of the extract and EICs of the four target compounds in the extract; (**C**) full-scan MS1 spectra of the four target compounds detected in the extract; (**D**) product-ion MS2 spectra of the four target compounds detected in the extract.

**Figure 8:**
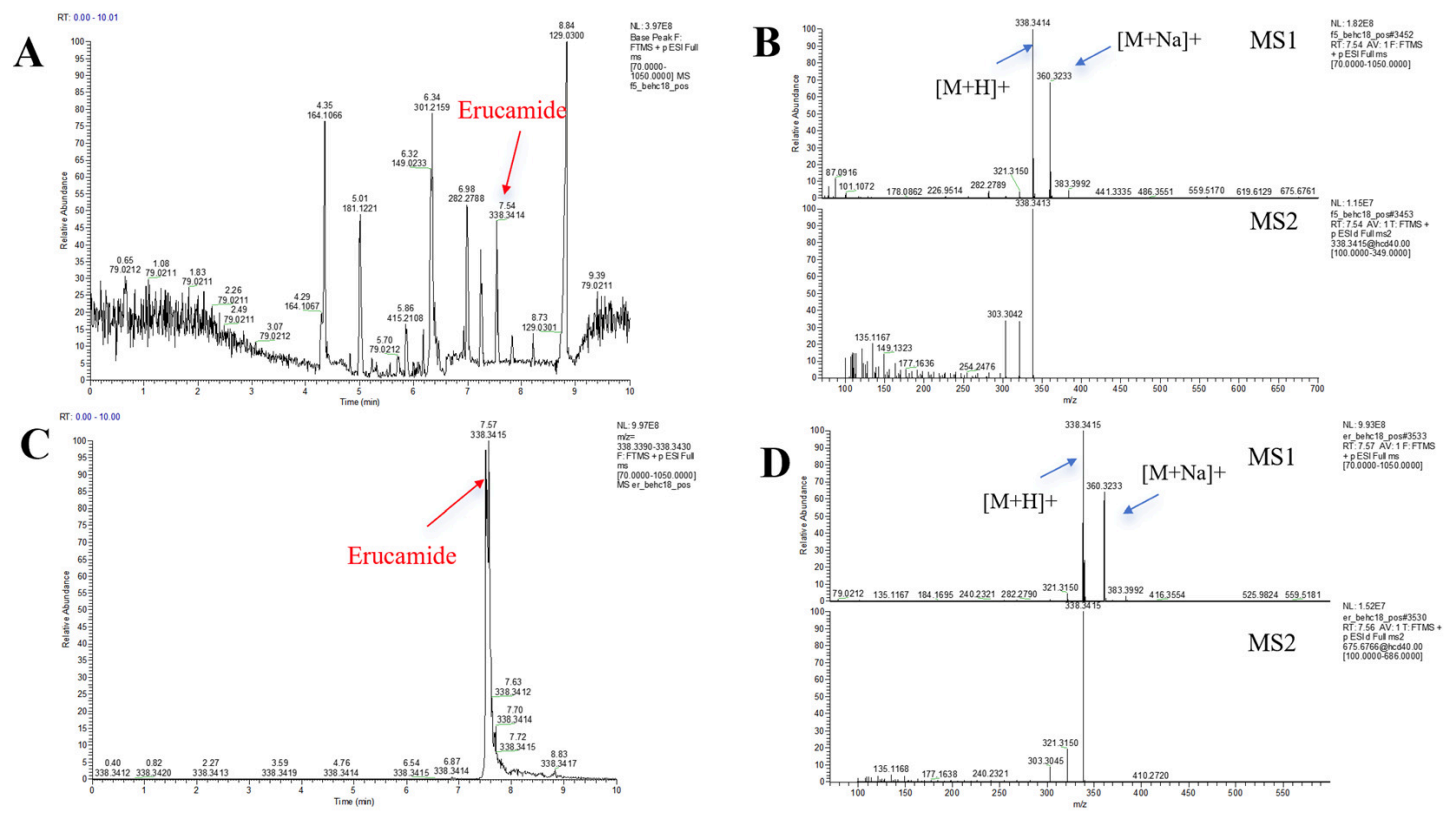
LC-MS/MS analysis results of erucamide standard and annotation results: (**A**) BPC chromatogram of the fraction F9; (**B**) MS1 and MS2 mass spectra of erucamide in F9; (**C**) EIC of erucamide standard; (**D**) MS1 and MS2 mass spectra of erucamide standard.

**Figure 9:**
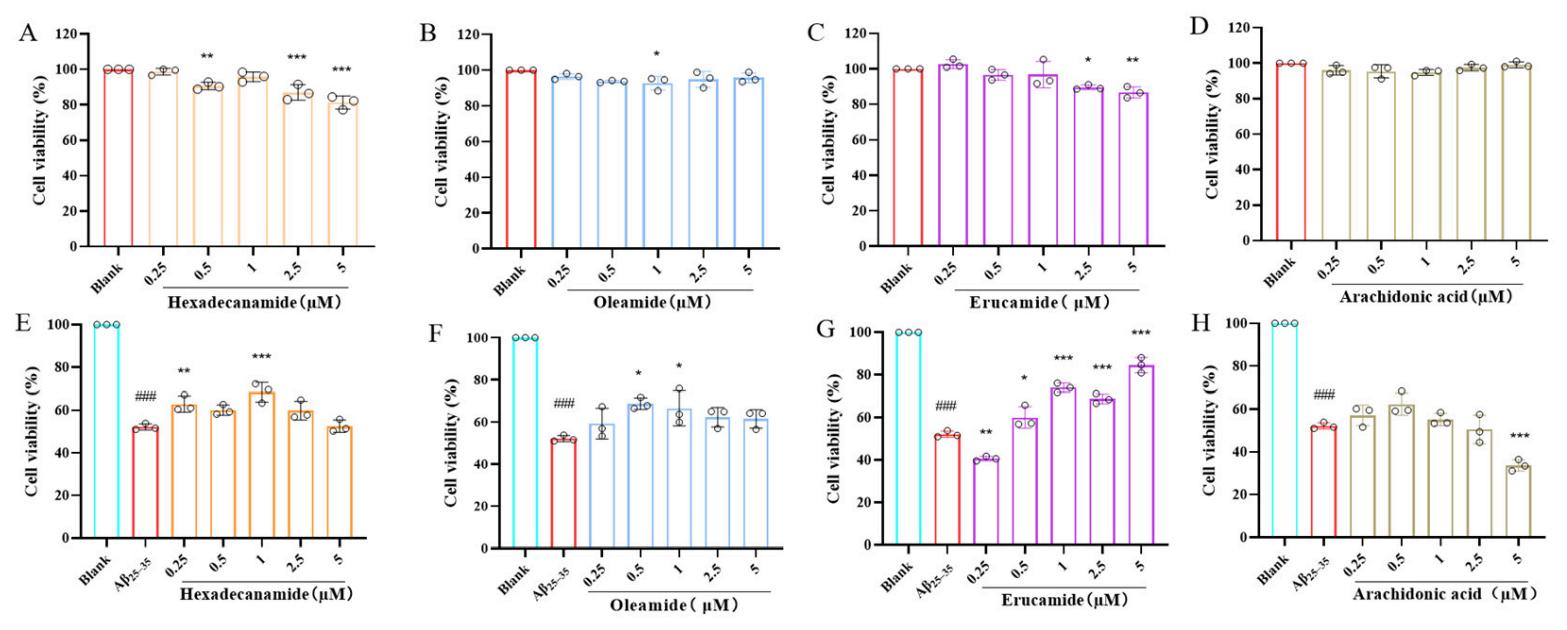
Effects of four annotated compounds on Aβ_25–35_-induced neuronal damage in HT-22 cells: (**A**–**D**) cell viability of HT-22 cells measured by MTT assay after treatment with different concentrations of four annotated compounds for 24 h; (**E**–**H**) cell viability of HT-22 cells measured by MTT assay after treatment with 20 µM Aβ_25–35_ for 24 h, followed by treatment with different concentrations of four annotated compounds for an additional 24 h. “#” indicates a significant difference between the model group and the blank control group (^###^ *p* < 0.001); “*” indicates a significant difference between the model group and the experimental group (* *p* < 0.05, ** *p* < 0.01, *** *p* < 0.001).

**Figure 10:**
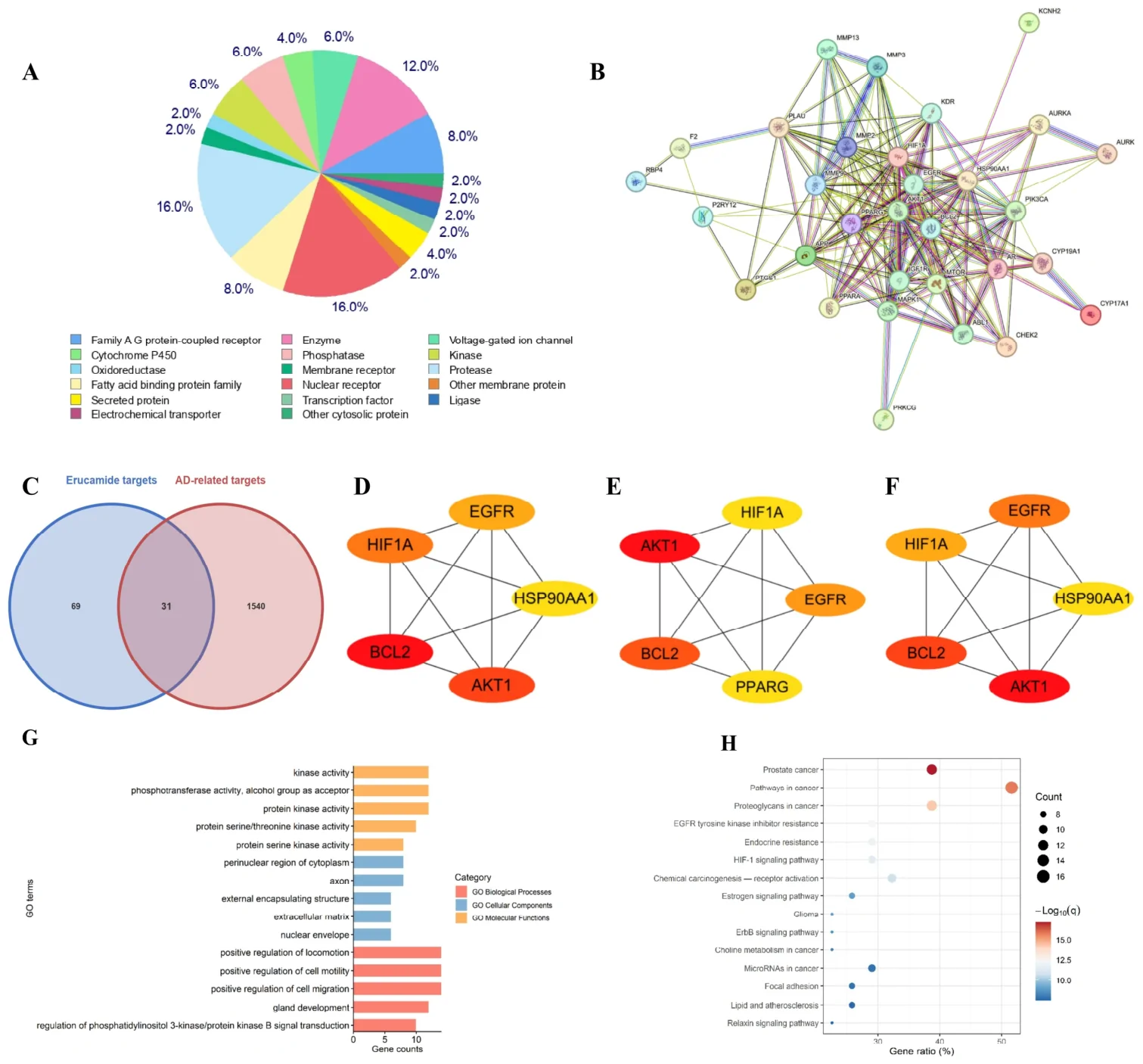
Network pharmacology prediction of the potential neuroprotective mechanism of erucamide: (**A**) classification of potential targets of erucamide; (**B**) protein–protein interaction (PPI) network of erucamide’s potential neuroprotective targets; (**C**) Venn diagram analysis of potential targets of erucamide and AD-related targets; (**D**–**F**) three topological analysis algorithms: MCC, Degree, and EPC; (**G**) GO analysis of neuroprotective targets of erucamide; (**H**) KEGG analysis of neuroprotective targets of erucamide.

**Figure 11:**
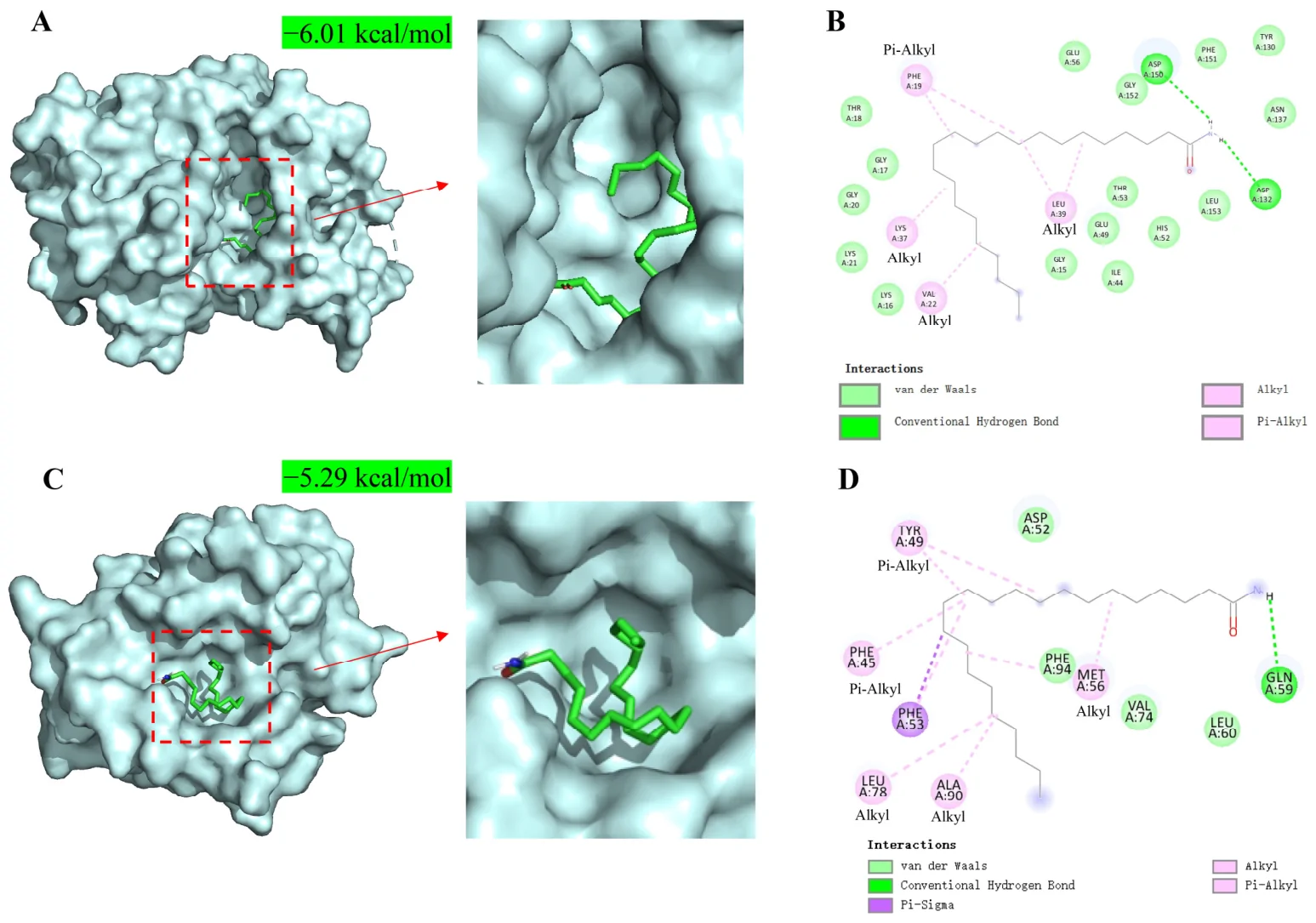
Molecular docking of erucamide with the target proteins: (**A**) 3D structure of the molecular docking between erucamide and the AKT1 protein; (**B**) 2D diagram of the interaction forces between erucamide and the AKT1 protein; (**C**) 3D structure of the molecular docking between erucamide and the BCL2 protein; (**D**) 2D diagram of the interaction forces between erucamide and the BCL2 protein.

**Figure 12:**
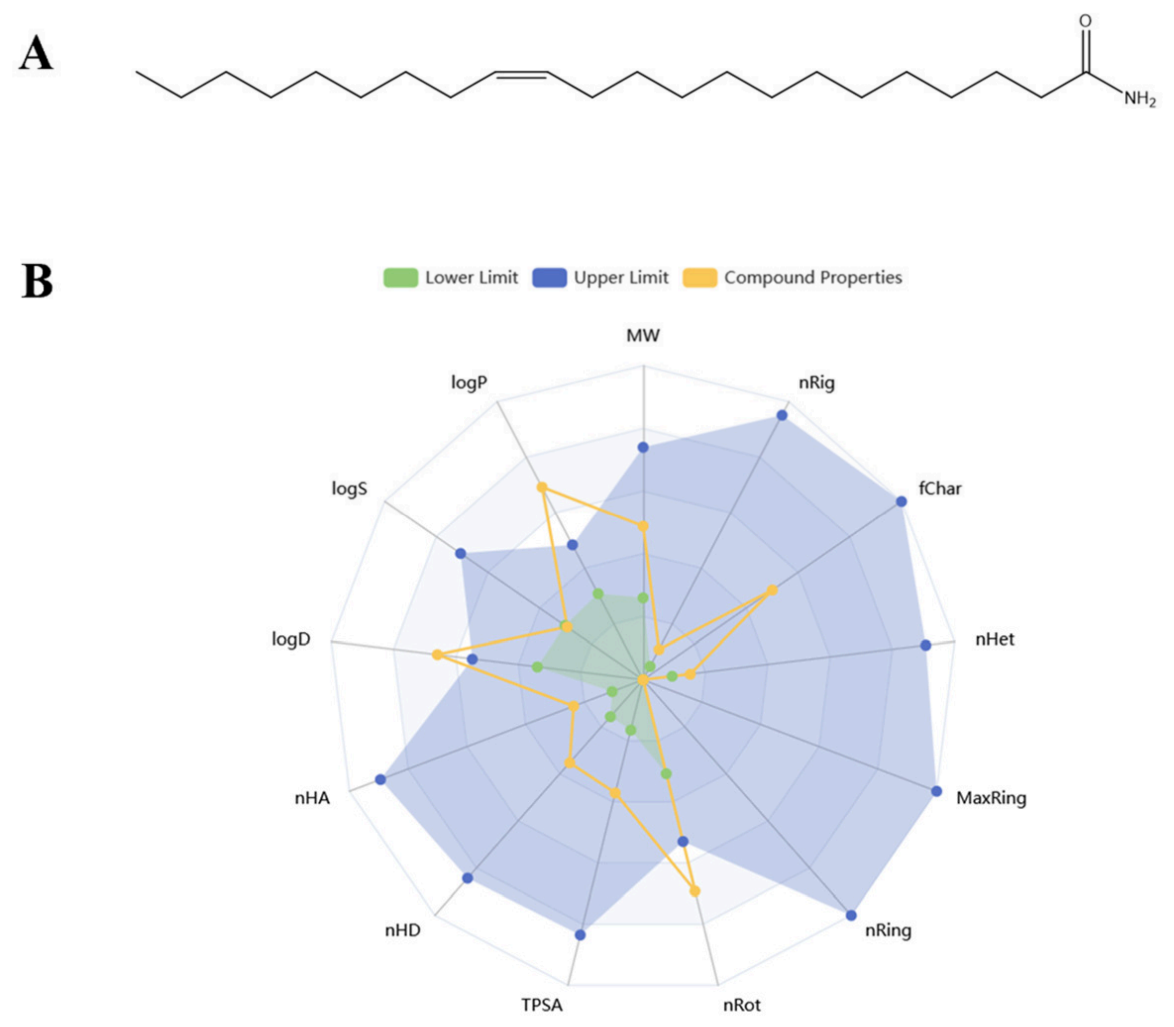
The chemical structure (**A**) and physicochemical properties prediction (**B**) of erucamide.

**Table 1:** Annotated results of potential neuroprotective compounds via Bio-LC-MS/MS combined with metabolomics tools.

Number	Compound 1	Compound 2	Compound 3	Compound 4
Compound name	Hexadecanamide	Oleamide	Erucamide	Arachidonic acid
Predicted formula	C_16_H_33_NO	C_18_H_35_NO	C_22_H_43_NO	C_20_H_32_O_2_
Average retention time (min)	6.962	7.009	7.553	7.227
Average (*m*/*z*)	256.2628	282.2784	338.3410	303.2332
Adduct ion name	[M + H] ^+^	[M + H] ^+^	[M + H] ^+^	[M−H] ^−^
Highest ion abundance (cps) in main fractions in positive mode †	641, 686, 845, 8 (F9) 568, 925, 744, 0 (F4) 559, 482, 317, 6 (F3)	394, 151, 094, 7 (F9) 195, 229, 350, 9 (F1) 175, 391, 932, 8 (F2)	992, 337, 654, 1 (F9) 628, 560, 566, 3 (F10) 322, 651, 604, 2 (F6)	170, 572, 103 (F2) 419, 200, 15 (F9) 126, 622, 9 (F10)
Cor *	0.87	0.44	0.47	0.82
Annotation method	MW-MF searching, MS/MS matching (MS-DIAL, MS-FINDER, GNPS)	MW-MF searching, MS/MS matching (MS-DIAL, MS-FINDER)	MW-MF searching, MS/MS matching (MS-DIAL, MS-FINDER)	MW-MF searching, MS/MS matching (MS-DIAL, MS-FINDER, GNPS)
Bioresource	Seaweed (*Galdieria sulphuraria* [37])	Seaweed (*Prymnesium parvum* [38])	Seaweed (*Ericaria* spp. [39])	Seaweed (*Gracilariopsis chorda* [40])

* Cor values were calculated using the bioactivity value at the dose of 2.5 μg/mL and MS data since the fractions showed larger differences in activity at this dose while no toxicity was observed. † For compound 4, negative mode was adopted.

**Table 2:** Drug-like properties and ADMET characteristics of erucamide.

Property	Value	Decision
Lipinski Rule	Accept (MW:337.33, Hdon:2)	Excellent
Blue Fluorescence	0.002	Excellent
Caco-2 Permeability	−5.123	Excellent
PAMPA	0.0	Excellent
Pgp-inhibitor	0.014	Excellent
Pgp-substrate	0.001	Excellent
Human Intestinal Absorption	0.004	Excellent
Volume Distribution	0.429	Excellent
Blood-Brain Barrier Penetration	0.056	Low
Drug-Induced Neurotoxicity	0.05	Excellent
Drug-Induced Liver Injury	0.007	Excellent

Note: Lipinski Rule: MW ≤ 500; logP ≤ 5; Hacc ≤ 10; Hdon ≤ 5. Two of the indicators out of range indicate that absorption or permeability may be poor, and one indicator out of range indicates that it is acceptable. Caco-2 Permeability: Higher than −5.15 Log unit. Blood-Brain Barrier Penetration: The numerical range is from 0 to 1, and the closer the value is to 1, the easier the compound passes through the blood-brain barrier, where 0–0.3 indicates low, 0.3–0.7 indicates moderate, and 0.7–1.0 indicates excellent. Human Intestinal Absorption: The numerical range is from 0 to 1, and the closer the value is to 1, the more difficult it is for the compound to be absorbed by the intestine, where 0–0.3 indicates excellent, 0.3–0.7 indicates medium, and 0.7–1.0 indicates poor.

## Data Availability

The raw MS data are deposited in Baidu Netdisk with folder name “raw data for Meicai Yue’s *Pterocladiella capillacea* manuscript published on Nutrients”. The permanently valid linkage is https://pan.baidu.com/s/1zwGyN0uaRFV6lm9KoBY-3A (accessed on 28 July 2026) with the code “jq8t”.

## Supplementary Materials

The following supporting information can be downloaded at https://www.mdpi.com/article/10.3390/nu18193210/s1: Figure S1: Detailed annotation of hexadecanamide. (A) MS2 spectral matching of the compound annotated by MSDIAL; (B) MS2 spectral matching of the compound annotated by MSFINDER; (C) MS2 image contrast diagram of GNPS annotated compounds; (D) Xcalibur-based analysis of MS1 data and precursor ion isotope pattern; (E) Isotope Distribution Calculator calculates the ionic molecular formula; (F) MS2 spectral matching of the compound annotated by MetFrag (in which the blue peaks represent the ones detected in measurement, the green ones represent the theoretical fragments based on structure, while the grey one represents the residue of precursor ion). Figure S2: Detailed annotation of oleamide. (A) MS2 spectral matching of the compound annotated by MSDIAL; (B) MS2 spectral matching of the compound annotated by MSFINDER; (C) Xcalibur-based analysis of MS1 data and precursor ion isotope pattern fidelity; (D) ChemDraw-simulated ion molecular formula calculation; (E) MS2 spectral matching of the compound annotated by MetFrag (in which the blue peaks represent the ones detected in measurement, the green ones represent the theoretical fragments based on structure, while the grey one represents the residue of precursor ion). Figure S3: Detailed annotation of arachidonic acid. (A) MS2 spectral matching of the compound annotated by MSDIAL; (B) MS2 spectral matching of the compound annotated by MSFINDER; (C) MS2 image contrast diagram of GNPS annotated compounds; (D) Xcalibur-based analysis of MS1 data and precursor ion isotope pattern fidelity; (E) ChemDraw-simulated ion molecular formula calculation; (F) MS2 spectral matching of the compound annotated by MetFrag (in which the blue peaks represent the ones detected in measurement, the green ones represent the theoretical fragments based on structure, while the grey one representds the residue of precursor ion). Figure S4: LC-MS/MS analysis results of hexadecanamide standard and annotation results. (A) BPC chromatogram of the fraction F9; (B) MS1 and MS2 mass spectra of hexadecanamide in F9; (C) EIC of hexadecanamide standard; (D) MS1 and MS2 mass spectra of hexadecanamide standard. Figure S5: LC-MS/MS analysis results of oleamide standard and annotation results. (A) BPC chromatogram of the fraction F9; (B) MS1 and MS2 mass spectra of oleamide in F9; (C) EIC of oleamide standard; (D) MS1 and MS2 mass spectra of oleamide standard. Figure S6: LC-MS/MS analysis results of arachidonic acid standard and annotation results. (A) BPC chromatogram of the fraction F9; (B) MS1 and MS2 mass spectra of arachidonic acid in F9; (C) EIC of arachidonic acid standard; (D) MS1 and MS2 mass spectra of arachidonic acid standard. Figure S7: Representative molecular networks of hexadecanamide and arachidonic acid in positive or negative ion modes. The color of the box indicates different ionization modes (positive/negative ion modes); node labels show compound names, m/z values, adduct ion types, compound structures, and correlation coefficients (cor values).

## Abbreviations

The following abbreviations are used in this manuscript:

AD	Alzheimer’s disease
*P. capillacea*	*Pterocladiella capillacea*
Aβ	Amyloid-β peptide
MTT	3-(4,5-dimethylthiazol-2-yl)-2,5-diphenyltetrazolium bromide
DPPH	2,2-Diphenyl-1-picrylhydrazyl
BCL2	B-cell lymphoma 2
AKT1	Protein kinase B
PBS	Phosphate-Buffered Saline
GNPS	Global Natural Products Social Molecular Networking
LC-MS/MS	Liquid Chromatography-Tandem Mass Spectrometry
Bio-LC-MS/MS	Bioactivity-coupled Liquid Chromatography-Tandem Mass Spectrometry
KEGG	Kyoto Encyclopedia of Genes and Genomes
GO	Gene Ontology
