# Supplementary material for: Neuroprotective Potential of the Edible Seaweed Pterocladiella capillacea: Bioactivity Coupled LC-MS/MS, Metabolomics Annotation, Validation, and Network Pharmacology
Source: Nutrients. 2026 Sep 29;18(19):3210. doi: 10.3390/nu18193210 (PMC13666746; doi:10.3390/nu18193210)
Supplement: Supplementary file 1 [file nutrients-18-03210-s001.zip › nutrients-4547385-supplementary.pdf]

Supplementary data

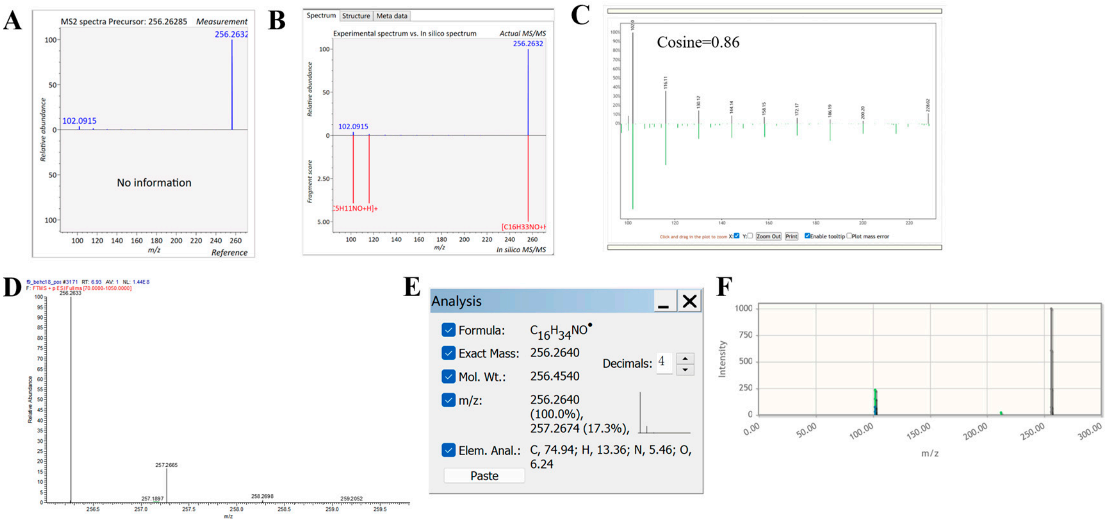

Figure S1. Detailed annotation of hexadecanamide. (A) MS2 spectral matching of the compound annotated by MSDIAL; (B) MS2 spectral matching of the compound annotated by MSFINDER; (C) MS2 image contrast diagram of GNPS annotated compounds; (D) Xcalibur-based analysis of MS1 data and precursor ion isotope pattern; (E) Isotope Distribution Calculator calculates the ionic molecular formula; (F) MS2 spectral matching of the compound annotated by MetFrag (in which the blue peaks represent the ones detected in measurement, the green ones represent the theoretical fragments based on structure, while the grey one represents the residue of precursor ion).

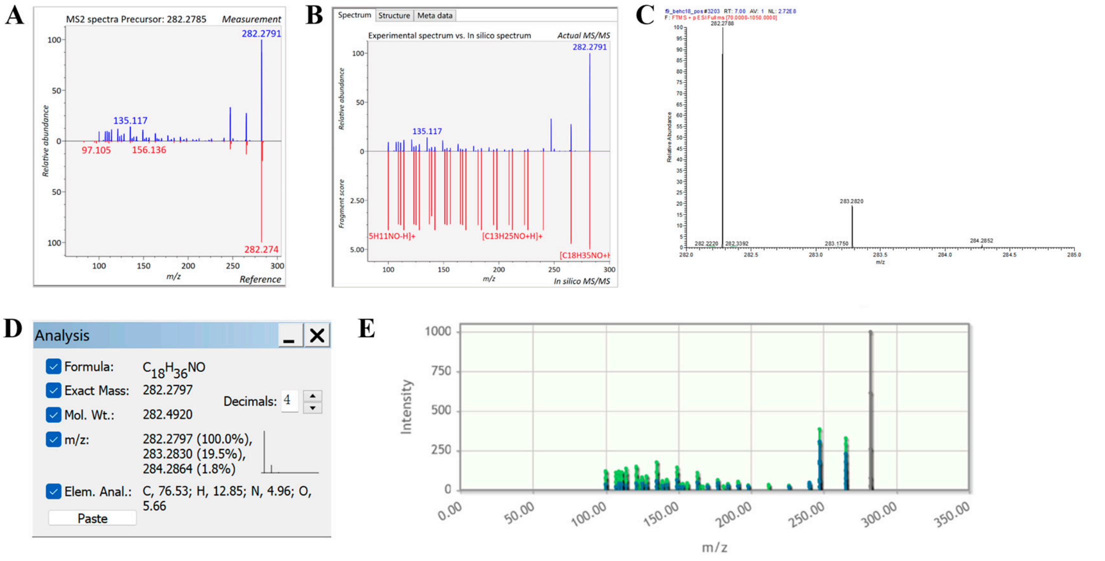

Figure S2. Detailed annotation of oleamide. (A) MS2 spectral matching of the compound annotated by MSDIAL; (B) MS2 spectral matching of the compound annotated by MSFINDER; (C) Xcalibur-based analysis of MS1 data and precursor ion isotope pattern fidelity; (D) ChemDraw-simulated ion molecular formula calculation; (E) MS2 spectral matching of the compound annotated by MetFrag (in which the blue peaks represent the ones detected in measurement, the green ones represent the theoretical fragments based on structure, while the grey one represents the residue of precursor ion).

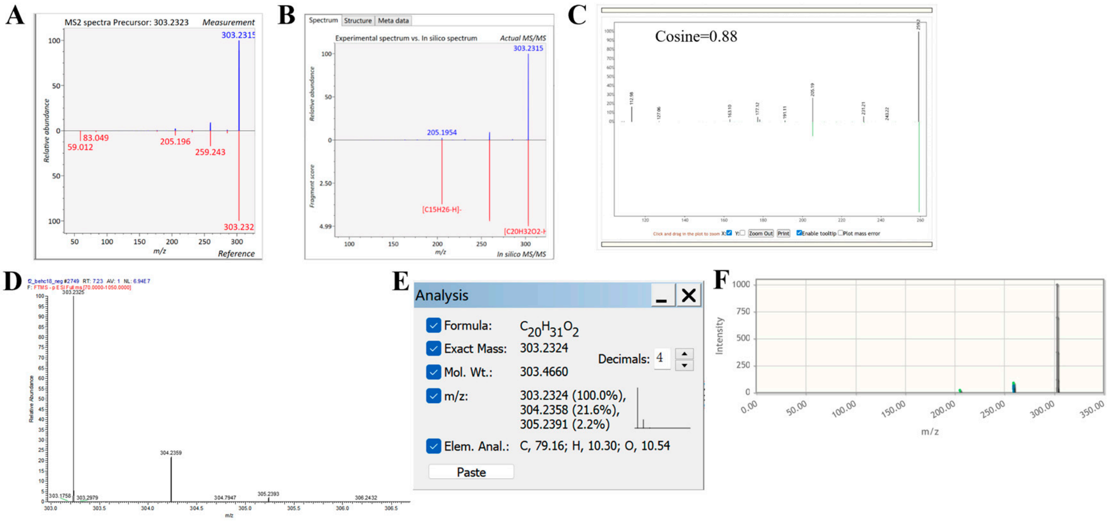

Figure S3. Detailed annotation of arachidonic acid. (A) MS2 spectral matching of the compound annotated by MSDIAL; (B) MS2 spectral matching of the compound annotated by MSFINDER; (C) MS2 image contrast diagram of GNPS annotated compounds; (D) Xcalibur-based analysis of MS1 data and precursor ion isotope pattern fidelity; (E) ChemDraw-simulated ion molecular formula calculation; (F) MS2 spectral matching of the compound annotated by MetFrag (in which the blue peaks represent the ones detected in measurement, the green ones represent the theoretical fragments based on structure, while the grey one representds the residue of precursor ion).

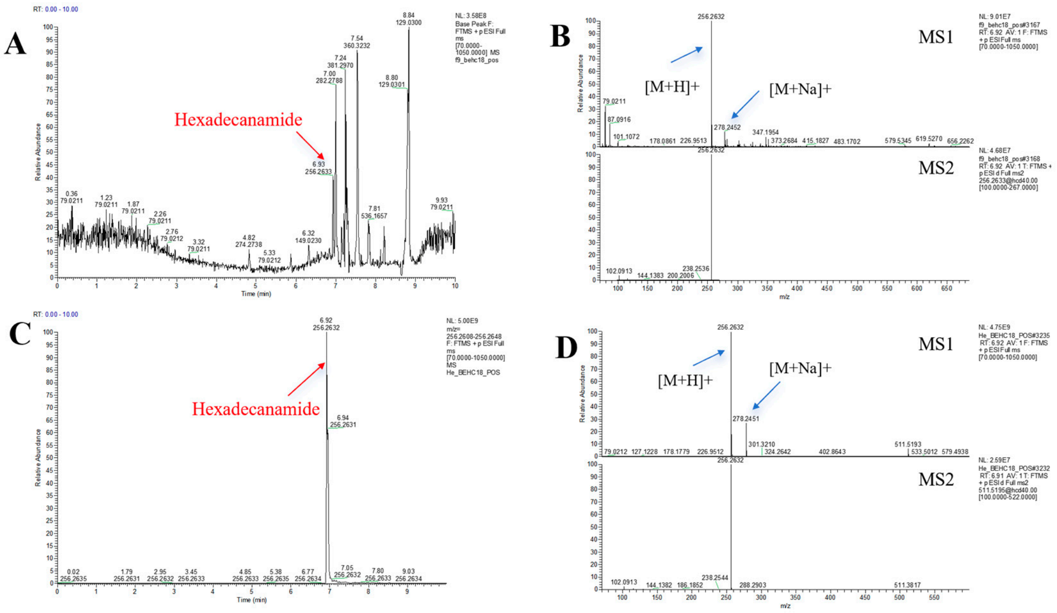

Figure S4. LC-MS/MS analysis results of hexadecanamide standard and annotation results. (A) BPC chromatogram of the fraction F9; (B) MS1 and MS2 mass spectra of hexadecanamide in F9; (C) EIC of hexadecanamide standard; (D) MS1 and MS2 mass spectra of hexadecanamide standard.

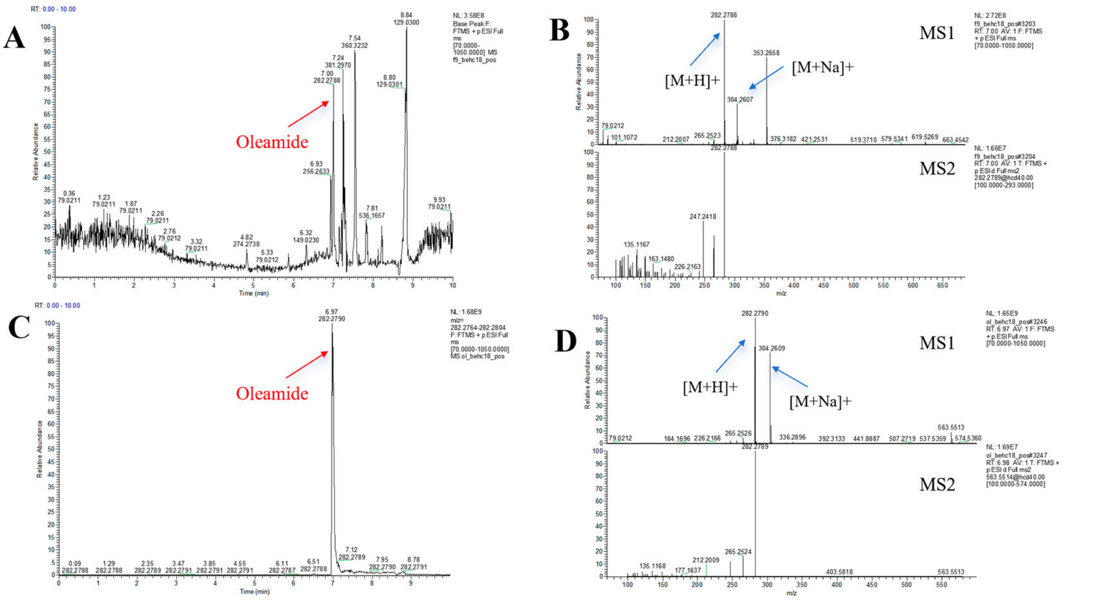

Figure S5. LC-MS/MS analysis results of oleamide standard and annotation results. (A) BPC chromatogram of the fraction F9; (B) MS1 and MS2 mass spectra of oleamide in F9; (C) EIC of oleamide standard; (D) MS1 and MS2 mass spectra of oleamide standard.

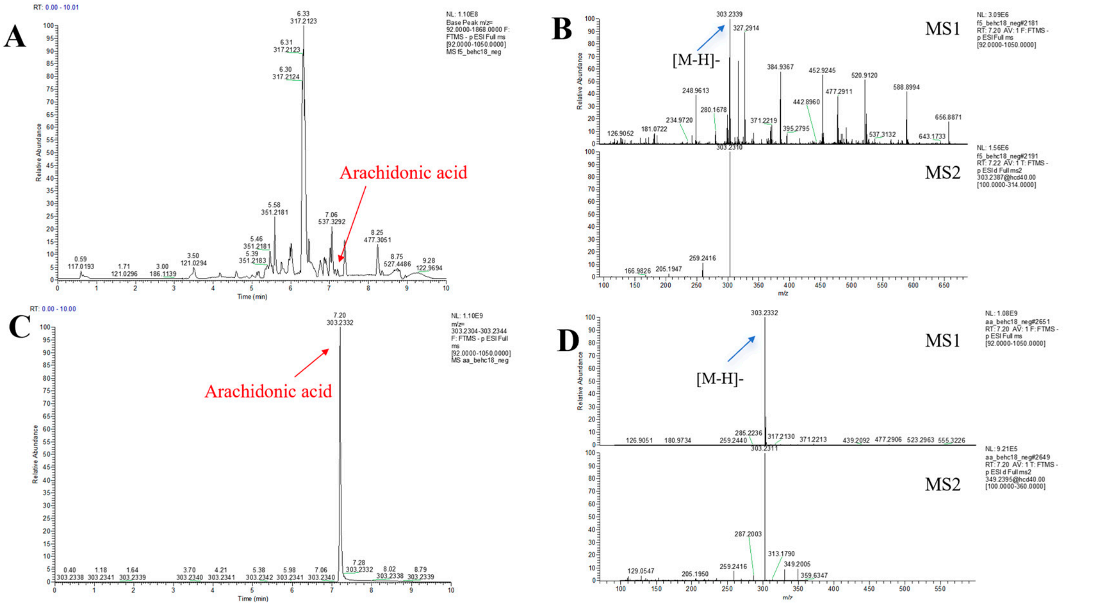

Figure S6. LC-MS/MS analysis results of arachidonic acid standard and annotation results. (A) BPC chromatogram of the fraction F9; (B) MS1 and MS2 mass spectra of arachidonic acid in F9; (C) EIC of arachidonic acid standard; (D) MS1 and MS2 mass spectra of arachidonic acid standard.

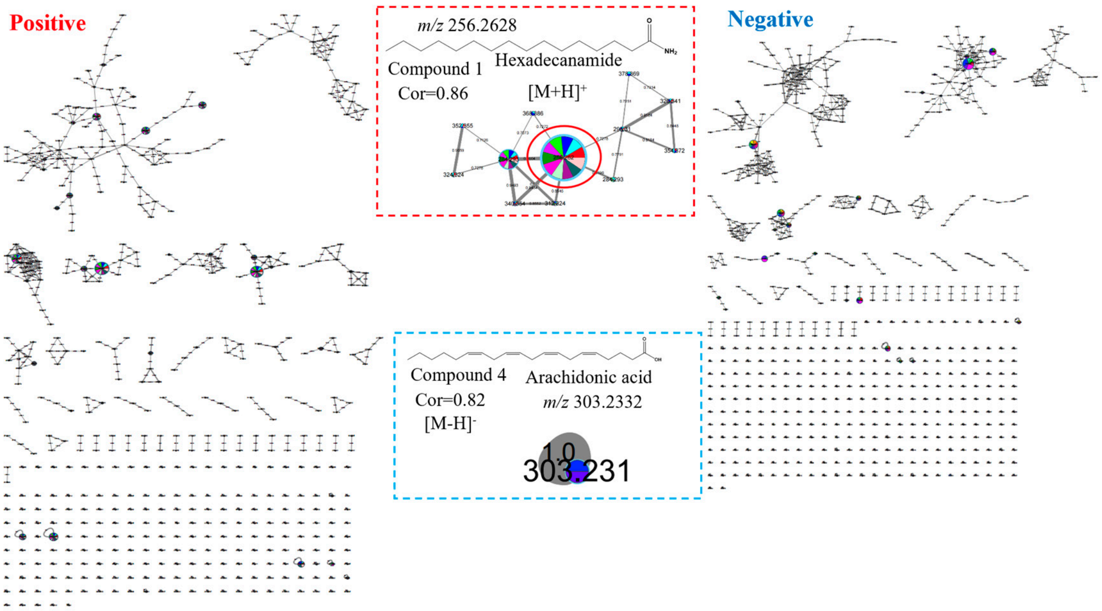

Figure S7. Representative molecular networks of hexadecanamide and arachidonic acid in positive or negative ion modes. The color of the box indicates different ionization modes (positive/negative ion modes); node labels show compound names, m/z values, adduct ion types, compound structures, and correlation coefficients (cor values).
